# Synaptotagmin-7–mediated activation of spontaneous NMDAR currents is disrupted in bipolar disorder susceptibility variants

**DOI:** 10.1371/journal.pbio.3001323

**Published:** 2021-07-06

**Authors:** Qiu-Wen Wang, Ying-Han Wang, Bing Wang, Yun Chen, Si-Yao Lu, Jun Yao

**Affiliations:** State Key Laboratory of Membrane Biology, Tsinghua-Peking Center for Life Sciences, IDG/McGovern Institute for Brain Research, School of Life Sciences, Tsinghua University, Beijing, China; Princeton University, UNITED STATES

## Abstract

Synaptotagmin-7 (Syt7) plays direct or redundant Ca^2+^ sensor roles in multiple forms of vesicle exocytosis in synapses. Here, we show that Syt7 is a redundant Ca^2+^ sensor with Syt1/Doc2 to drive spontaneous glutamate release, which functions uniquely to activate the postsynaptic GluN2B-containing NMDARs that significantly contribute to mental illness. In mouse hippocampal neurons lacking Syt1/Doc2, Syt7 inactivation largely diminishes spontaneous release. Using 2 approaches, including measuring Ca^2+^ dose response and substituting extracellular Ca^2+^ with Sr^2+^, we detect that Syt7 directly triggers spontaneous release via its Ca^2+^ binding motif to activate GluN2B-NMDARs. Furthermore, modifying the localization of Syt7 in the active zone still allows Syt7 to drive spontaneous release, but the GluN2B-NMDAR activity is abolished. Finally, Syt7 SNPs identified in bipolar disorder patients destroy the function of Syt7 in spontaneous release in patient iPSC-derived and mouse hippocampal neurons. Therefore, Syt7 could contribute to neuropsychiatric disorders through driving spontaneous glutamate release.

## Abstract 2

Synaptotagmin-7 (Syt7) variants are associated with susceptibility to bipolar disorder; this study shows that Syt7 acts as a calcium sensor to drive spontaneous glutamate release which uniquely activates postsynaptic GluN2B-containing glutamate receptors. Interestingly, this function of Syt7 is disrupted in bipolar disorder susceptibility variants.

## Introduction

Neurotransmitter release is driven by an increase in intracellular Ca^2+^. Three types of Ca^2+^-dependent release have been identified: Action potential (AP) evoked fast synchronous release, slow asynchronous release, and AP-independent spontaneous release [1,2]. All these types are triggered by the orchestration of the SNARE complex, consisting of the v-SNARE synaptobrevin-2 (Syb2), the t-SNAREs syntaxin-1A (Stx1a) and SNAP-25, and their respective Ca^2+^ sensor proteins [3–10]. The synaptotagmins are a family of proteins that contain a transmembrane domain and 2 tandom C2 domains called C2A and C2B. Synaptotagmin-1 (Syt1), Syt2, and Syt9 have been suggested to be the Ca^2+^ sensors for fast synchronous release, and the mechanisms of synaptic vesicle (SV) fusion have been elucidated during the past decade through investigating the interaction between Syt1 and the SNARE complex [6,11,12]. The Ca^2+^ sensor family for the slow asynchronous release contains 2 players, Doc2 and Syt7 [7,8]. Doc2 is a cytosolic protein containing 2 C2 domains without a transmembrane domain [13,14] and likely contacts most SVs that are tightly docked or primed at the active zone (AZ) membrane. Unlike Doc2, Syt7 likely interplays only with a small subpopulation of readily releasable SVs due to its restricted distribution in the peripheral AZ [15]. Regarding the spontaneous release, at present, it has been found that at least 2 proteins, Syt1 and Doc2, can drive this mode of release [9,16]. Therefore, it seems that spontaneous release might be a heterogeneous phenomenon involving mechanisms partially overlapping with synchronous and asynchronous release.

Syt7 is a high-affinity Ca^2+^ sensor that interacts with low concentration Ca^2+^, SNAREs, and phospholipids to induce robust vesicle fusion with slow kinetics [17]. In presynaptic terminals, Syt7 has been found to drive asynchronous SV release [8,18]. Syt7 can also contribute to short-term synaptic plasticity, such as synaptic facilitation and SV replenishment [19,20], probably through its sensitive responses to the incremental residual Ca^2+^ in the bouton. Furthermore, Syt7 can mediate the slow phase of SV endocytosis [21], suggesting that Syt7 is important for the coupling of the exo- and endocytosis of SVs. In addition, in the postsynaptic density (PSD), Syt7 and Syt1 are likely redundant Ca^2+^ sensors for the trafficking of AMPA receptors (AMPARs) and thus can regulate long-term potentiation (LTP) [22]. Recently, we have found that Syt7 is a susceptible gene for bipolar disorder (BD) and that its deficits could induce bipolar-like behaviors in mice [15,23]. Syt7 is localized to the peripheral AZ membrane and triggers AP-evoked glutamate release to activate the juxtaposed postsynaptic GluN2B-containing NMDARs, the deficits of which underlie a mechanism for the behavioral abnormalities in Syt7-deficient animals.

In the present study, using the Syt7 knockout (KO) mouse model and CRISPR interference (CRISPRi)-based gene knockdown (KD) technique, we investigated the role of Syt7 in spontaneous SV release. We found that Syt7 acted as a redundant Ca^2+^ sensor to drive spontaneous glutamate release in mouse hippocampal neurons, which functioned uniquely to activate the juxtaposed postsynaptic GluN2B-NMDARs. Furthermore, modifying the localization of Syt7 in the AZ still allowed Syt7 to trigger spontaneous release, but the positional noncorrespondence abolished the activation of the GluN2B-NMDARs. Finally, we showed that Syt7 single nucleotide polymorphisms (SNPs) identified in BD patients could not activate GluN2B-NMDARs through spontaneous release in BD patient induced pluripotent stem cell (iPSC)-derived or mouse hippocampal neurons. We therefore concluded that Syt7 was a compelling candidate Ca^2+^ sensor for a subpopulation of spontaneous release events with a unique physiological function that distinguished them from those triggered by Syt1/Doc2.

## Results

### Syt7 is a redundant Ca^2+^ sensor for spontaneous release in hippocampal neurons

**Fig 1:**
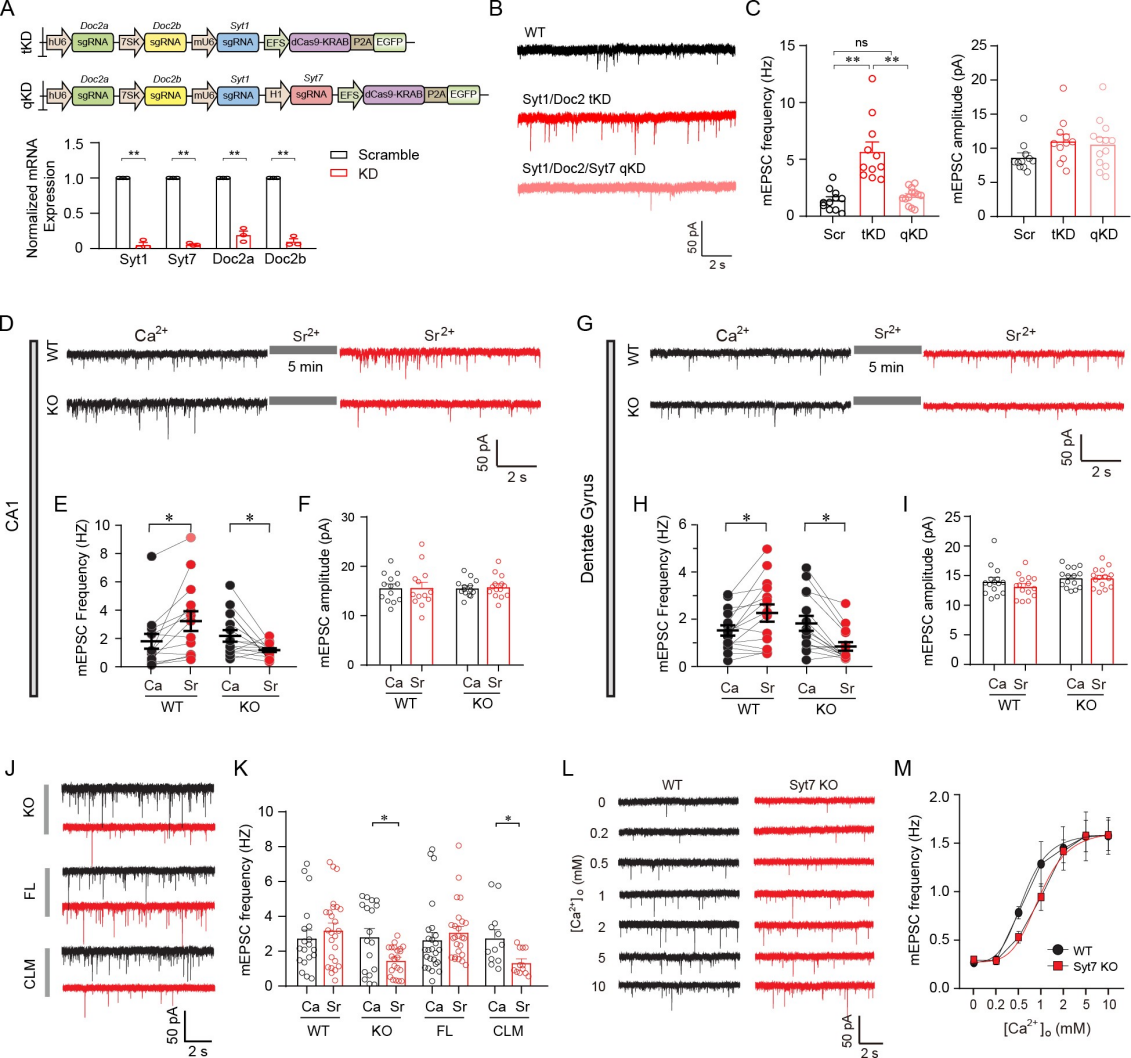
Syt7 triggers spontaneous neurotransmitter release. (**A**) Design of multiplex CRISPRi system. Upper, schematic illustration of the multiple gene targeting CRISPRi for Syt1/Doc2a/2b and Syt1/Syt7/Doc2a/2b. Lower, qRT-PCR analysis of mRNA expression of hippocampal neurons with multiplex KD of Syt1/Syt7/Doc2a/2b. (**B**) Sample traces of mEPSCs recorded in cultured hippocampal neurons with multiplex gene KD. (**C**) Quantification of the frequency (left) and amplitude (right) of mEPSCs. WT, *n =* 11; tKD, *n* = 11; qKD, *n* = 13. (**D**) Representative traces of mEPSCs recorded from hippocampal CA1 slices. The same neuron was recorded in the presence of extracellular Ca^2+^ or Sr^2+^ in the ACSF solution with a 5-min interval. (**E**) Paired comparison of the frequency of Ca^2+^- and Sr^2+^-triggered mEPSCs in hippocampal CA1 slices. WT, *n =* 13; KO, *n* = 14. (**F**) Bar graph of mEPSC amplitude. (**G–I**) Sample traces (**G**) and quantification of frequency (**H**) and amplitude (**I**) of mEPSCs recorded in the DG slices. WT, *n* = 15; KO, *n* = 15. (**J**) Representative traces of Ca^2+^- or Sr^2+^-triggered mEPSCs recorded from cultured WT hippocampal neurons, Syt7 KO neurons, and KO neurons expressing Syt7^FL^ or Syt7^CLM^. (**K**) Quantitative analysis of the frequency of mEPSCs. WT, *n =* 19 for Ca^2+^, *n* = 25 for Sr^2+^; Syt7 KO, *n* = 18/21; KO + Syt7^FL^, *n* = 25/25; KO + Syt7^CLM^, *n* = 12/12. (**L**) Representative traces of mEPSCs recorded from WT and Syt7 KO neurons in a Ca^2+^ gradient from 0 mM to 10 mM. (**M**) Quantitative analysis of mEPSC frequency recorded in Ca^2+^ gradient. Red curves are fitted Hill function. Ca^2+^ affinity: WT, 1.52 ± 0.53; KO, 1.35 ± 0.52; *P* = 0.819. Ca^2+^ cooperativity: WT, 0.46 ± 0.11; KO, 0.77 ± 0.24; *P* = 0.258. WT, *n =* 13–19; Syt7 KO, *n* = 16–20. Student *t* test; *P < 0.05; **P < 0.001; error bars, SEM. The numerical data underlying this figure are included in S1 Data. CLM, calcium ligand mutant; CRISPRi, CRISPR interference; DG, dentate gyrus; FL, full-length; KD, knockdown; KO, knockout; mEPSC, miniature excitatory postsynaptic current; qKD, quadra KD; qRT-PCR, quantitative reverse transcription PCR; Syt7, synaptotagmin-7; tKD, triple KD; WT, wild-type.

Syt1 and Doc2 have been suggested to be Ca^2+^ sensors for spontaneous SV release [9,16]. Previously, it has been suggested that Syt7 and Syt1 were redundant Ca2+ sensors for postsynaptic AMPAR trafficking that underlie an important mechanism for the induction of LTP [22]. Moreover, the robust spontaneous release in Syt1-deficient neurons has suggested that spontaneous release is likely triggered by redundant mechanisms [16]. Although it has been reported that Syt7 KO hippocampal neurons have no apparent changes in the rate of spontaneous release [19], we were interested to know whether Syt7 could be a redundant Ca^2+^ sensor with Syt1 and Doc2. To this end, we designed a CRISPRi-based gene KD system to achieve multiplex inactivation of Ca^2+^ sensors, including Syt1, Doc2a, Doc2b, and Syt7 (**Fig 1A**). We expressed the CRISPRi system in cultured wild-type (WT) hippocampal neurons through lentiviral infection. Quantitative reverse transcription PCR (qRT-PCR) analysis verified that the mRNA expression of these 4 genes was largely abolished. We evaluated the spontaneous SV release by analyzing the AMPAR-mediated miniature excitatory postsynaptic currents (mEPSCs) (**Fig 1B**). The results indicated that compared to the scrambled neurons, the Syt1/Doc2a/Doc2b triple KD (tKD) neurons showed an increase in the frequency of mEPSCs; however, compared to the tKD group, neurons with an addition of Syt7 KD (Syt1/Doc2a/Doc2b/Syt7 quadra KD (qKD)) showed a reduced mEPSC frequency, which was similar to the level of the scrambled neurons (**Fig 1C**). Moreover, the mEPSC amplitude was unaffected by the KD of genes. These results indicated that Syt7 could trigger spontaneous SV release in the absence of Syt1 and Doc2 and thus was a redundant Ca^2+^ sensor for spontaneous glutamate release.

Previously, it has been suggested that unlike most Syt isoforms including Syt1, Syt7 can interact with Sr^2+^ to trigger membrane fusion in vitro [17]. Hence, based on a previously reported Syt7 KO mouse model in which the C2A domain was replaced with a neomycin cassette [24], we employed Sr^2+^ as a tool to analyze the role of Syt7 in spontaneous release in the hippocampal CA1 slices (**Fig 1D**). Following the mEPSC recordings in the presence of 2 mM Ca^2+^, the extracellular Ca^2+^ was replaced with Sr^2+^. After 5 min, a second recording was performed in the same neuron in the presence of Sr^2+^. Recordings in the same WT CA1 neuron revealed an increase in the mEPSC frequency when Ca^2+^ was replaced by Sr^2+^, whereas in the Syt7 KO neurons, the mEPSC frequency was decreased in response to the Ca^2+^/Sr^2+^ transition (**Fig 1E**). However, the mEPSC amplitude was unchanged (**Fig 1F**). We repeated the patch clamp recording analysis in the dentate gyrus (DG) neurons of the hippocampal slices and observed similar results (**Fig 1G–1I**).

To test whether Syt7 triggered spontaneous release through its Ca^2+^ binding motif, we expressed the Ca^2+^ ligand mutant (Asp225 and 227 in C2A, Asp357 and 359 in C2B, to Asn) of Syt7 (Syt7^CLM^), which abolished the Ca^2+^ binding activity of Syt7, in the cultured hippocampal neurons through lentiviral introduction (**Fig 1J**). We found that unlike hippocampal slices, the cultured WT neurons did not show any significant changes in mEPSC frequency when Ca^2+^ was switched to Sr^2+^ (**Fig 1K**). However, consistent with the results of the slice recording, in the Syt7 KO neurons, Sr^2+^ resulted in a reduction in mEPSC frequency. We noted that in cultured neurons lacking Syt1 or Doc2, the Ca^2+^/Sr^2+^ transition did not induce any obvious changes in mEPSC frequency (**S1 Fig**). Hence, the Sr^2+^-induced reduction in mEPSC frequency in the Syt7 KO neurons and slices was directly caused by the loss of Syt7. Importantly, this reduction could be rescued by the WT full-length Syt7 (Syt7^FL^) but not by Syt7^CLM^ (**Fig 1K**). These results indicated that Syt7 could directly trigger spontaneous SV release through its Ca^2+^ binding motif. In addition, we compared the Ca^2+^/Sr^2+^-triggered mEPSCs in the cultured hippocampal neurons with Syt1 KD and Syt1/Syt7 double KD (dKD) and confirmed that the role of Syt7 in spontaneous release was unaffected by the presence of Syt1 (**S2 Fig**).

Furthermore, we investigated the mEPSCs driven by Ca^2+^ concentration gradients (in mM: 0, 0.2, 0.5, 1, 2, 5, and 10) in the hippocampal CA1 slices of Syt7 KO mice (**Fig 1L**). We found that compared to the WT group, the Ca^2+^ sensitivity curve showed a right shift in the Syt7 KO neurons, although the analyses of Ca^2+^ cooperativity and affinity did not show statistically significant differences between the WT and Syt7 KO neurons (**Fig 1M**). Hence, these results indicated that Syt7 probably played a physiological role in spontaneous release.

Together, our results indicated that Syt7 was able to function as a redundant Ca^2+^ sensor to trigger spontaneous glutamate release in mouse hippocampal neurons.

### Syt7-triggered spontaneous glutamate release activates GluN2B-NMDARs

**Fig 2:**
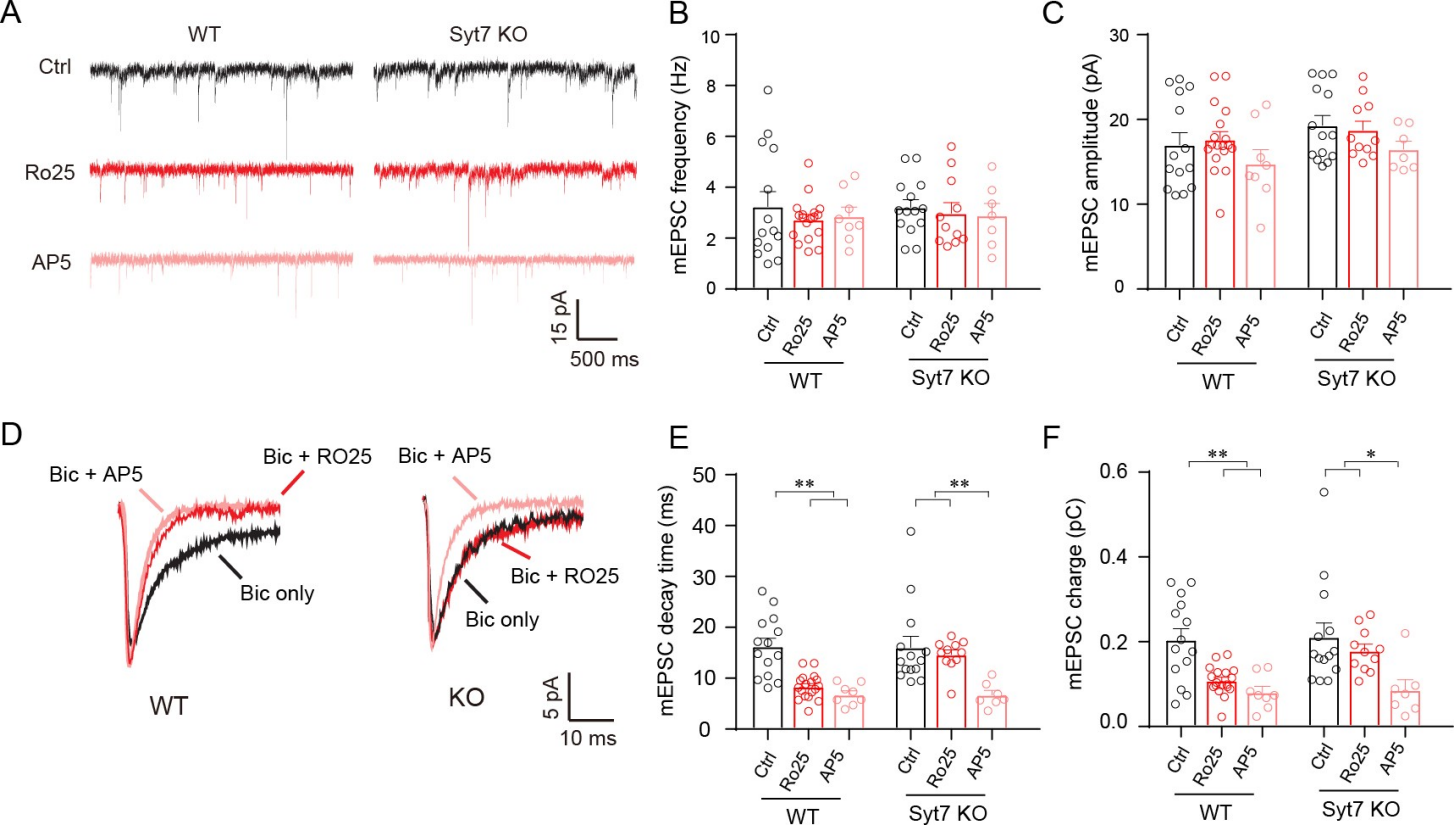
Syt7 triggered spontaneous glutamate release specifically activates GluN2B-NMDARs. (**A**) Sample traces of AMPAR/NMDAR-mEPSCs in hippocampal slices of WT and Syt7 KO neurons. (**B, C**) Bar graphs summarizing the frequency (**B**) and amplitude (**C**) of AMPAR/NMDAR-mEPSCs. (**D**) Average traces of AMPAR/NMDAR-mEPSCs. (**E, F**) Bar graphs summarizing the decay time (**E**) and total charge (**F**). (**A–F**) WT, *n =* 14/18/8; KO, *n* = 14/11/7. Student *t* test; *P < 0.05; **P < 0.001; error bars, SEM. The numerical data underlying this figure are included in S1 Data. AMPAR, AMPA receptor; KO, knockout; mEPSC, miniature excitatory postsynaptic current; Syt7, synaptotagmin-7; WT, wild-type.

We then asked why the synapses employed so many Ca^2+^ sensors to drive spontaneous release; namely, did these diverse subtypes of release have different physiological functions? Recently, we have found that Syt7 KO mice showed bipolar-like behavioral abnormalities through a mechanism involving the hypoactivity of GluN2B-NMDARs [15,23]. Hence, we performed electrophysiological recordings on the hippocampal slices of the WT and Syt7 KO mice to test whether Syt7-triggered spontaneous glutamate release could activate the GluN2B-mediated NMDARs compared to the other 2 Ca^2+^ sensors (**Fig 2A**). To facilitate the recognition of NMDAR-mEPSC events, we generated the co-occurrence of AMPAR activation during the recordings and analyzed the AMPAR/NMDAR-mEPSCs. We applied Ro25-6981, a GluN2B-specific antagonist, to the recording chamber to evaluate the GluN2B activity; AP5 was used to evaluate all NMDAR activity. We found that in both the WT and Syt7 KO neurons, the application of Ro25-6981 did not induce any obvious changes in the frequency or amplitude of the AMPAR/NMDAR-mEPSCs (**Fig 2B and 2C**). When we analyzed the charge and kinetics of the mEPSCs (**Fig 2D**), we found that in the WT neurons, the Ro25-6981 treatment could significantly reduce the decay time and charge transfer compared to the untreated control neurons (**Fig 2E and 2F**). This result indicated that in the WT neurons, spontaneous glutamate release could efficiently activate GluN2B-NMDARs. In contrast, in the Syt7 KO slices, we observed that the acute application of Ro25-6981 failed to significantly affect the decay time or charge transfer of the AMPAR/NMDAR-mEPSCs, which could still be reduced by AP5 (**Fig 2E and 2F**). This result indicated that in the Syt7 KO mice, where the spontaneous release was predominantly triggered by Syt1/Doc2, the GluN2B activity was attenuated during spontaneous neurotransmission. We also tested the GluN2B activity in cultured hippocampal neurons with Syt1 KD or Syt1/Syt7 dKD and observed that the Ro25-6981-induced attenuation of GluN2B activity was detected in the Syt1 KD neurons but not in the Syt1/Syt7 dKD neurons (**S3 Fig**), indicating that Syt7 could trigger spontaneous GluN2B activity when Syt1 was absent.

We then investigated whether this attenuation was caused by the absence or mislocalization of GluN2B-NMDARs at postsynaptic spines. Immonoblot and qRT-PCR analyses revealed that the expression of GluN2B was up-regulated in the Syt7 KO neurons compared to the WT controls (**S4A Fig**). Moreover, the previously reported developmental switch of NMDARs from GluN2B to GluN2A was not affected by the Syt7 deficiency (**S4B Fig**) [25,26]. We also carried out super-resolution stochastic optical reconstruction microscopy (STORM) analysis and observed that the Syt7 deficiency did not affect the synaptic distribution of GluN2B in the hippocampal neurons (**S4C and S4D Fig**). These observations are consistent with our previous study [15]. We therefore concluded that Syt7-triggered spontaneous glutamate release was an important source of GluN2B activation in the mouse brain, which distinguished it from that triggered by Syt1 or Doc2.

### Retargeting Syt7 to the central AZ triggers non-GluN2B spontaneous transmission

**Fig 3:**
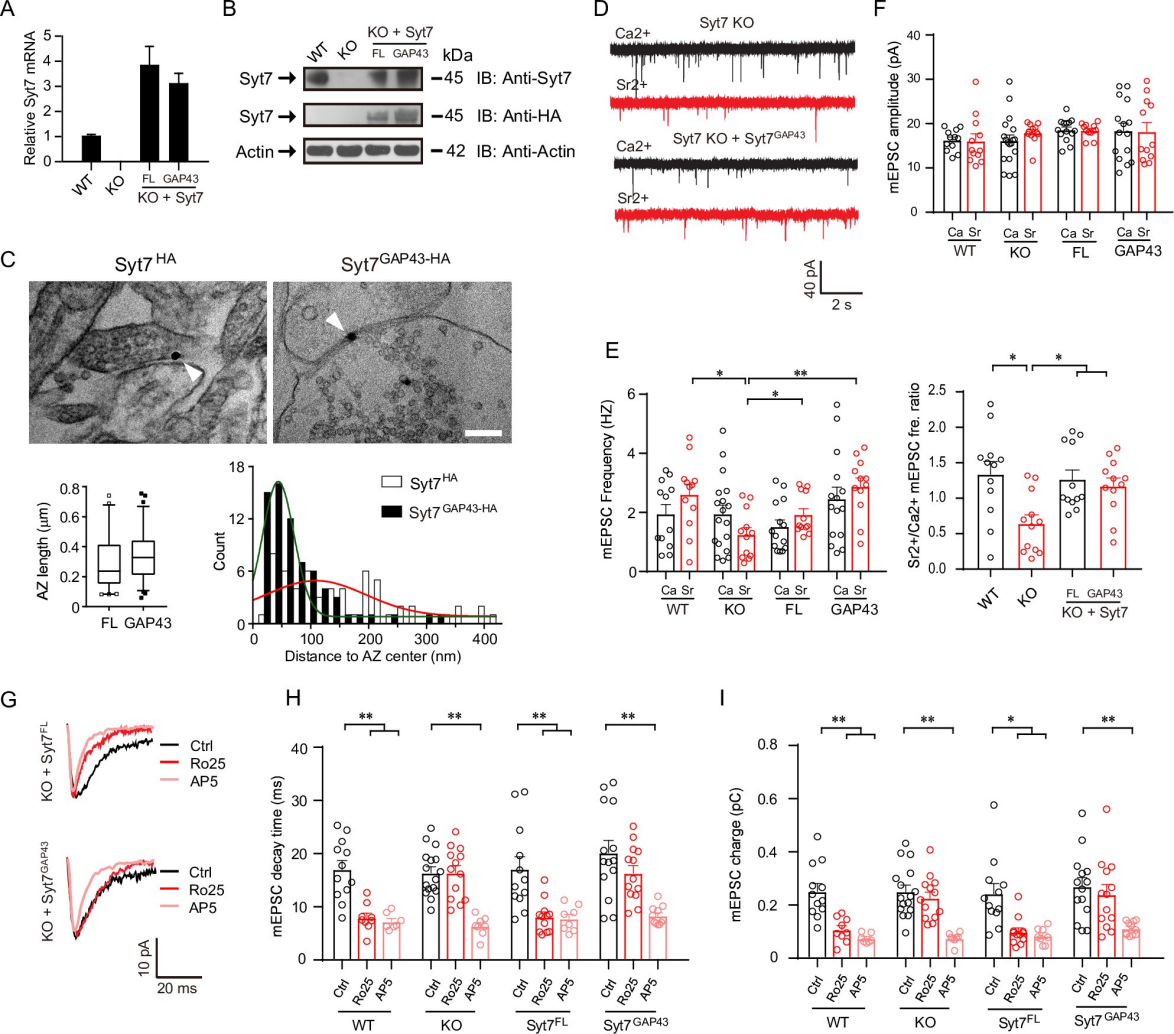
Retargeting Syt7 to the central AZ triggers non-GluN2B spontaneous neurotransmission. (**A, B**) qRT-PCR (**A**) and IB (**B**) analyses showing the lentiviral expression of HA-conjugated Syt7 in neurons. (**C**) EM analysis showing that Syt7^GAP43^-HA (*n =* 69) was closer to the center of AZ compared to Syt7^FL^-HA (*n* = 59). Upper, sample EM images. Scale bar, 200 nm. Lower left, AZ length. Lower right, histogram of Syt7^FL^-HA/Syt7^GAP43^-HA distribution. Red and green curves are fitted Gaussian curve. (**D**) Sample traces of Ca^2+^/Sr^2+^-triggered mEPSCs in WT neurons (*n* = 12/12), Syt7 KO neurons (*n* = 18/12), and KO neurons expressing Syt7^FL^ (*n* = 14/12) or Syt7^GAP43^ (*n* = 15/12). (**E**) Analysis of mEPSC frequency (left) and Sr^2+^/Ca^2+^ ratio of frequency (right). WT, *n* = 12/12; KO, *n* = 18/12; Syt7^FL^, *n* = 14/12; Syt7^GAP43^, *n* = 15/12. (**F**) mEPSC amplitude. (**G**) Average traces showing the kinetics of AMPAR/NMDAR-mEPSCs in Syt7^GAP43^-expressing neurons following GluN2B blockade. (**H, I**) Decay time (**H**) and total charge (**I**) of mEPSCs. WT, *n* = 12/9/7; KO, *n* = 16/13/8; Syt7^FL^, *n* = 12/12/8; Syt7^GAP43^, *n* = 14/13/11. ANOVA (see S6 Fig); *P < 0.05; **P < 0.001; error bars, SEM. The numerical data underlying this figure are included in S1 Data. AMPAR, AMPA receptor; AZ, active zone; EM, electron microscopy; FL, full-length; IB, immunoblot; KO, knockout; mEPSC, miniature excitatory postsynaptic current; qRT-PCR, quantitative reverse transcription PCR; Syt7, synaptotagmin-7; WT, wild-type.

Previously, we have demonstrated that Syt7 and GluN2B were localized to the peripheral synaptic region, with the former in the peripheral AZ membrane and the latter in the peripheral PSD [15]. To help understand the role of Syt7 in spontaneous SV release, we employed a protein chimera Syt7^GAP43^, in which the C2 domains of Syt7 were conjugated to a short N-terminus 20 amino acid peptide of GAP43, to retarget Syt7 to the whole AZ. We carried out immunoelectron microscopy (immuno-EM) analysis to compare the localization of Syt7 and Syt7^GAP43^ in the presynaptic AZ membrane. As the commercial Syt7 antibodies cannot label Syt7^GAP43^, we added an HA tag to the C-terminus of WT full length Syt7 (Syt7^FL^-HA) or Syt7^GAP43^ (Syt7^GAP43^-HA) to facilitate the immuno-EM experiments. qRT-PCR and immunoblot analyses revealed that HA-conjugated Syt7 was expressed in the hippocampal neurons and could be detected by the HA-specific antibody (**Fig 3A and 3B**).

We then carried out EM analysis using the HA antibody. We observed that the Syt7^FL^-HA and Syt7^GAP43^-HA groups of neurons showed a similar AZ length of 250 to 300 μm (**Fig 3C**). Moreover, Syt7^GAP43^-HA was largely present within a distance of 150 μm from the AZ center, whereas the distribution curve of Syt7^FL^-HA was shifted to the outer region (**Fig 3C**). Hence, Syt7^FL^-HA was largely absent from the central AZ, while Syt7^GAP43^-HA was retargeted to the whole AZ. We also evaluated the distribution of Syt7 at the global level using an immunofluorescence-based Duolink in situ proximity ligation assay (PLA) (**S5A Fig**). Compared to the association of Stx1a to SNAP-25, the Syt7-SNAP-25 Duolink puncta showed lower fluorescence density and smaller area, indicating that Syt7 had very limited contact overall with SNAP-25 compared to Stx1a (**S5B and S5C Fig**). We then compared the DUOlink puncta of Syt7^FL^-HA and Syt7^GAP43^-HA and observed that Syt7^GAP43^-HA showed much greater contact with the SNARE proteins (**S5D and S5E Fig**), indicating that Syt7^GAP43^-HA was widely distributed in the AZ.

Next, we carried out patch clamp recording experiments to analyze the effects of Syt7^GAP43^ on AMPAR-mediated mEPSCs (**Fig 3D**). We employed the acute Ca^2+^/Sr^2+^ transition protocol and found that similar to our previous observation, the Syt7 KO neurons showed an obvious reduction in mEPSC frequency in response to the Ca^2+^/Sr^2+^ switch; moreover, this reduction was rescued by both Syt7^FL^ and Syt7^GAP43^ (**Figs 3E and S6A**). Hence, Syt7^GAP43^ was able to trigger spontaneous release. We noted that the mEPSC amplitude was unchanged in all groups (**Figs 3F and S6B**). We then assayed the GluN2B activity during the Syt7^GAP43^-triggered AMPAR/NMDAR-mEPSCs (**Fig 3G**). We observed that the Ro25-6981 treatment induced an obvious attenuation in the decay time and charge transfer of the mEPSCs in the WT neurons but not the Syt7 KO neurons (**Figs 3H and 3I and S6C and S6D**), which is consistent with our previous observations. Importantly, the GluN2B hypoactivity in the KO neurons was rescued by Syt7^FL^, but it could not be significantly changed by Syt7^GAP43^, indicating that Syt7^GAP43^ could not function to effectively activate the GluN2B-NMDARs. In addition, in all groups of neurons, the Ro25-6981 treatment did not affect the frequency; however, it led to a decrease in the mEPSC amplitude when Syt7 or Syt7^FL^ was present (**S6E and S6F Fig**).

Together, our results showed that modifying the localization of Syt7 in the AZ still allowed Syt7 to drive spontaneous release, but the positional noncorrespondence largely diminished the activation of GluN2B-NMDARs, which supported the unique function of Syt7-triggered release.

### Human Syt7 variants induce GluN2B hypoactivity in human iPSC-derived and mouse hippocampal neurons

**Fig 4:**
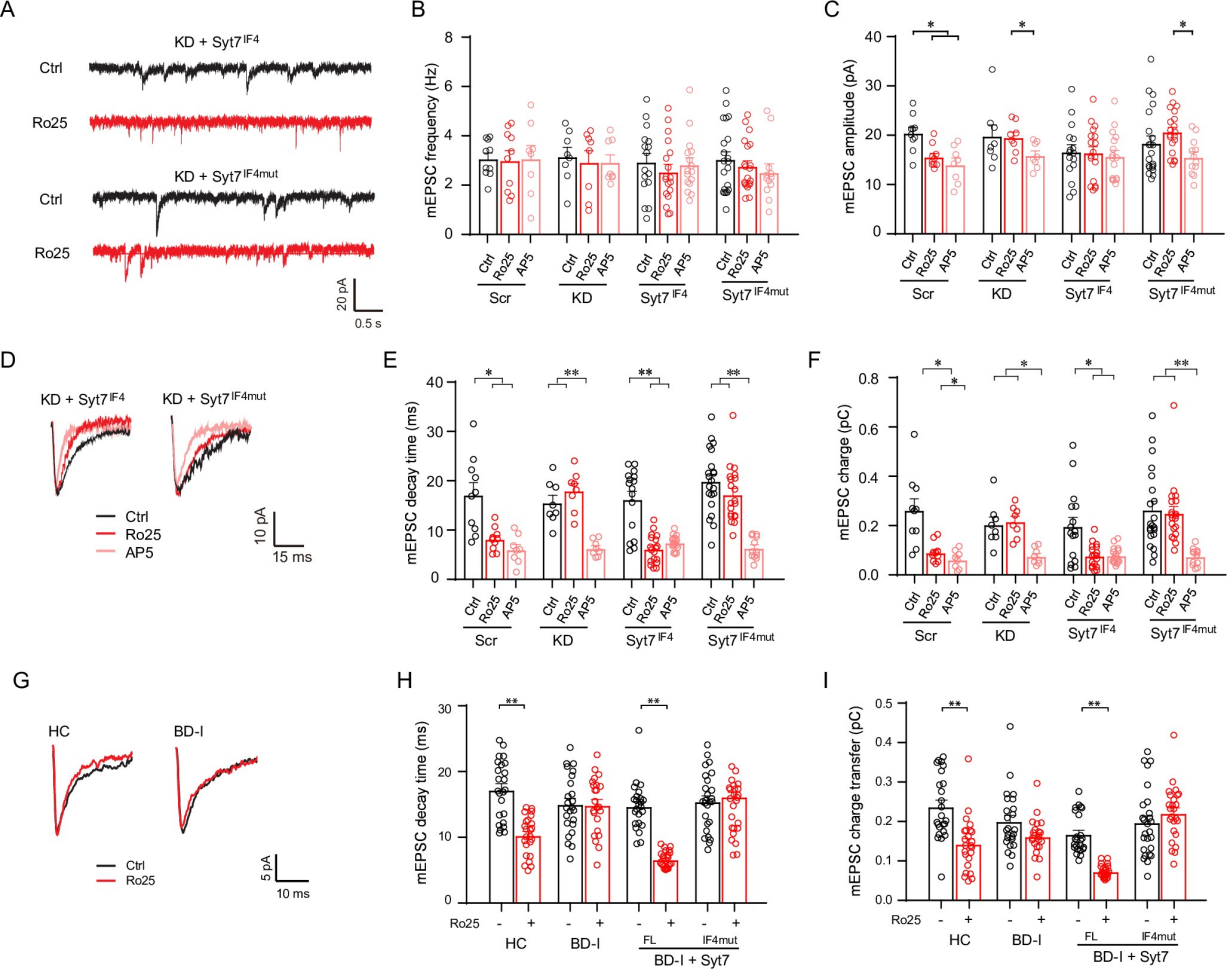
Human Syt7 variants induce deficits in spontaneous glutamate release and GluN2B activity. (**A**) Sample traces of AMPAR/NMDAR-mEPSCs in Syt7 KD neurons expressing Syt7^IF4^ and Syt7^IF4mut^. (**B, C**) Bar graphs summarizing the frequency (**B**) and amplitude (**C**) of mEPSCs. Scr, *n =* 10/10/8; Syt7 KD, *n* = 8/8/8; Syt7 ^IF4^, *n* = 16/18/16; Syt7^IF4mut^, *n* = 21/20/12. (**D**) Average traces of AMPAR/NMDAR-mEPSCs. (**E, F**) Bar graphs summarizing the decay time (**E**) and total charge (**F**). (**G**) Average traces showing changes in the kinetics of AMPAR/NMDAR-mEPSCs in the iPSC-derived DG-like neurons of healthy controls and BD-I patients. (**H, I**) Bar graphs summarizing the decay time (**H**) and charge transfer (**I**) of AMPAR/NMDAR-mEPSCs in the HC neurons, BD-I neurons, and BD-I neurons overexpressing Syt7^FL^ or Syt7^IF4mut^. For all groups, *n* = 23–26 neurons of 3 cell lines. Student *t* test; *P < 0.05; **P < 0.001; error bars, SEM. The numerical data underlying this figure are included in S1 Data. AMPAR, AMPA receptor; BD-I, bipolar disorder I; DG, dentate gyrus; HC, healthy control; iPSC, induced pluripotent stem cell; KD, knockdown; mEPSC, miniature excitatory postsynaptic current; Syt7, synaptotagmin-7.

Previously, we have identified several Syt7 SNPs in BD patients and demonstrated that the Syt7 gene was associated with susceptibility to this disease [15]. Moreover, the Syt7 SNPs, L227M and Q247H, could attenuate the Syt7-triggered asynchronous and short-term plasticity forms of glutamate release. Both mutations resided on exon 6 of the human *SYT7* gene, which encodes the juxtamembrane linker region of the Syt7 protein and showed damage potential on the structure of the Syt7 protein by Harmfulness evaluation analysis with the Polyphen2_HDIV and Polyphen2_HVAR algorithms. In the mice, Syt7 isoform 4 had these 2 mutation sites. Thus, we compared the performances of isoform 4 of mouse Syt7 (Syt7^IF4^) and its L152M/Q172H mutant corresponding to human L227M/Q247H (Syt7^IF4mut^) in spontaneous release. In cultured WT neurons expressing the Syt7-specific CRISPRi rather than Syt7 KO neurons, we expressed Syt7^IF4^ and Syt7^IF4mut^ through lentiviral infection and investigated the spontaneous GluN2B activity (**Fig 4A**). Analysis of AMPAR/NMDAR-mEPSCs revealed that compared to the scrambled neurons, the Syt7 KD neurons and KD neurons expressing Syt7^IF4^ or Syt7^IF4mut^ showed similar frequencies and amplitudes of mEPSCs; moreover, the Ro25-6981 treatment did not affect either the mEPSC frequency or amplitude in these 2 groups of neurons, whereas AP5 could reduce the mEPSC amplitude (**Fig 4B and 4C**). Consistent with our observations in the Syt7 KO neurons, the decay time and charge transfer of AMPAR/NMDAR-mEPSCs were unaffected by Ro25-6981 in the Syt7 KD neurons. Importantly, Ro25-6981 could significantly reduce the decay time and charge transfer of the mEPSCs in the Syt7^IF4^-expressing KD neurons, but it failed to affect the Syt7^IF4mut^-expressing KD neurons (**Fig 4D–4F**). Therefore, in mouse hippocampal neurons, the 2 Syt7 SNPs could not efficiently activate GluN2B-NMDARs via spontaneous glutamate release.

We then aimed to ascertain whether the deficits in spontaneous release existed in the BD patient neurons, which have insufficient Syt7 expression [23]. To this end, we analyzed the AMPAR/NMDAR-mEPSCs in the hippocampal DG-like neurons derived from the iPSCs of 3 sporadic BD-I patients and 3 healthy controls [27], as well as patient neurons with lentiviral overexpression of Syt7^FL^ or Syt7^IF4mut^. The neurons differentiated from the iPSCs were mostly Prox1^+^ DG granule cells and showed normal AP firing (**S7A and S7B Fig**). The results indicated that the decay time and the charge transfer of the mEPSCs were not significantly different between the 4 groups (**Figs 4G–4I and S7C–S7J**). The Ro25-6981 treatment induced an obvious reduction in the mEPSC decay time and charge transfer of the healthy control neurons, whereas the diseased neurons were only slightly affected. Moreover, the diseased neurons overexpressing Syt7^FL^ showed a significantly reduced decay time or charge transfer in response to the Ro25-6981 treatment, whereas the neurons overexpressing Syt7^IF4mut^ failed to change the slight response of the diseased neurons to the drug treatment. These results indicated that the hippocampal neurons derived from the iPSCs of these BD patients showed Syt7 deficit induced GluN2B dysfunction and could be restored by the exogenous introduction of Syt7 but not by the Syt7 SNPs of BD patients.

Together, our data indicated that Syt7 deficit-induced defects in spontaneous release could induce GluN2B dysfunctions, which might contribute to the etiology of the investigated BD cases.

## Discussion

Candidate Ca^2+^ sensors for spontaneous release included Syt1, Doc2, and Syt7. The former two have been verified to function to drive this type of release [9,16]. Recently, it has been reported that Syt7 could participate in a pathological stress-induced augmentation of spontaneous release [28]. In the present study, we demonstrated that Syt7 acted as a redundant Ca^2+^ sensor to trigger spontaneous glutamate release that contributed to the activation of postsynaptic GluN2B-NMDARs. The physiological relevance of Syt7 to spontaneous release has long been unrecognized, because the mEPSC frequency failed to show changes in the Syt7-deficient neurons or in the Syt1/Syt7 double-deficient neurons [8,19]. In recent years, accumulating evidence has revealed that Ca^2+^-dependent vesicle exocytosis can have redundant mechanisms. For instance, Syt1 and Syt7 are redundant Ca^2+^ sensors that mediate AMPAR exocytosis during LTP [22]. In Syt1-deficient neurons, it seemed that at least one sensor with greater Ca^2+^ binding affinity worked to induce the enhancement of the spontaneous release. However, it has been found that simply inactivating Doc2 or Syt7 alone in neurons lacking Syt1 could not address the redundancy of spontaneous release [8,16,29]. In the present study, we utilized the multiplex CRISPRi technique to abolish the expression of all 3 candidate Ca^2+^ sensors, including Syt1, Syt7, and Doc2a/2b, and found that the redundantly enhanced spontaneous release in Syt1-deficient neurons could be largely diminished. This result indicated that Syt7 and Doc2 probably played co-redundant roles in triggering spontaneous release in the Syt1-deficient neurons. We note that the occurrence of spontaneous release events is often regulated by AP firing-relevant homeostatic plasticity [30]. Hence, it is necessary to consider the factors involving chronic homeostasis, in addition to redundancy, when evaluating the function of AP-related proteins in spontaneous release. This is supported by the fact that Syt1 drives a majority of the AP-triggered SV release, and in Syt1-deficient neurons, the rate of spontaneous release is increased. As Doc2 could affect asynchronous release in neurons, which contributes to neural network excitability and synaptic plasticity, it is also possible that Doc2 can indirectly affect the mEPSC frequency through modulating the baseline neural network activity. In the present study, we employed an acute Ca^2+^/Sr^2+^ switching approach and demonstrated that Syt7 could have an instant impact on the rate of spontaneous release. Previously, in vitro reconstitution studies have revealed that Syt7 could efficiently interact with Sr^2+^ to induce robust membrane fusion, whereas Syt1 and most other Syt isoforms could not [17]. In physiological tests, it was found that Sr^2+^ could promote AP evoked asynchronous SV release [31,32]. Particularly, even in the Syt1-deficent neurons, the application of extracellular Sr^2+^ would attenuate the fast synchronous phase of SV release, while the asynchronous phase release remained unaffected compared to the application of Ca^2+^, leading to a deceleration in the decay of the overall SV release [33]. All these findings support that compared to Ca^2+^, Sr^2+^ has a weakened interaction with Syt1 and other synchronous Ca^2+^ sensors (e.g., Syt2) to induce fast SV release, while the asynchronous release remains unaffected. Hence, it was appropriate to employ Sr^2+^ as a tool to compare the roles of Syt7 and Syt1 in spontaneous release. Moreover, the Syt7 KO neurons showed alterations in the Ca^2+^ sensitivity of the spontaneous SV release. In addition, we confirmed that the Syt7 deficiency- or mutant-induced changes in mEPSC frequency were not caused by abnormalities in the synapse density, readily releasable SV pool (RRP) size or protein mislocalization (**S8 Fig**). These results, together with previous findings [9,16], indicated that spontaneous release is a heterogeneous phenomenon involving multiple mechanisms that can have redundant and homeostatic adjustment, and Syt7 is one of the Ca^2+^ sensors involved. We note that the Sr^2+^ application increased the mEPSC frequency in the WT slices but not in the WT neuronal cultures (**Fig 1**). This discrepancy could be caused by several reasons. First, the protein machinery for SV fusion might have some differences in the brain slices and cultured neurons (e.g., different splice variants of SNAREs) and thus have slightly different Sr^2+^ response kinetics. Second, the intracellular Sr^2+^ buffering system might have some differences in the brain slices and cultured neurons, and thus the intracellular Sr^2+^ concentration might be different.

Our results indicated that the Syt7-triggered spontaneous release has a unique function compared to that triggered by Syt1 or Doc2, that it could be an important source for the activity of GluN2B-NMDARs in the brain. It has been widely shown that GluN2B is likely an important NMDAR subunit involved in mental illnesses including BD. Importantly, this dysfunction was not only induced by the Syt7 deficiency but was also shown by the BD susceptibility variants of Syt7. In the present study, the role of Syt7 in GluN2B activation was further verified by the Syt7^GAP43^ mutant experiments, which targeted Syt7 to the broad AZ. Previously, it has been suggested that posttranslational modifications in the N-terminal segment, which could direct the protein trafficking and participate in scaffolding interactions, were the determinants for the different trafficking routes of Syt7 and Syt1 [34]. Using Syt7^GAP43^ and Syt7^GAP43^-HA, we not only removed the peripheral AZ trafficking potential of Syt7 but also endowed Syt7 with the ability to broadly distribute in the AZ.

Conventionally, it has been thought that NMDARs are likely saturated by quantal size spontaneous glutamate release, based on the electrophysiological analysis of NMDAR current kinetics [35]. However, this opinion has been challenged in the past more than 10 years. For instance, it has been suggested that in the postsynaptic region, spontaneous and evoked release may activate different populations of NMDARs [36], leading to differential responses to ketamine, the prominent NMDAR-targeted antidepressant [37]. These findings provide direct evidence that NMDARs were unlikely to be saturated by spontaneous release. Here, our data provided further evidence to support that separated subpopulations of NMDARs showed correspondence to different presynaptic SV pools.

Finally, we note that in the current study, the mEPSC amplitude analysis showed discrepant results in our experiments. For instance, in **Figs 2C** and **4C**, Ro25-6981 and AP5 did not affect the mEPSC amplitude; however, in **Figs 4C** and **S2B**, these 2 drugs could induce decreases in the mEPSC amplitude. There are 2 possible reasons for this discrepancy. First, the experiments in **Fig 2** were performed on brain slices, while those in **Figs 4** and **S2** were performed on cultured neurons. In different neuronal systems, the rising kinetics of NMDAR-mEPSC might have some differences, which might be sufficient to affect the mEPSC amplitude when combined with AMPAR-mEPSCs. Second, as the time period for our study spanned several years and several generations of animals were used for the electrophysiological recordings in these figures, the later offspring could show accumulative impacts of some unknown epigenetic modifications. In this case, the functional phenotype of Syt7 in spontaneous release persisted throughout these animal generations, indicating that Syt7 is a compelling candidate Ca^2+^ sensor for spontaneous release.

## Materials and methods

### Animals and plasmids

Syt7 KO mice were kindly provided by E.R. Chapman (Madison, Wisconsin, USA) with permission from N.W. Andrews (College Park, Maryland, USA). All the animal experiments were conducted under the guidance and with the approval of the Institutional Animal Care & Use Committee of Tsinghua University and the Animal Welfare and Ethics Committee of Tsinghua University (Ethics approval number: F16-00228; A5061-01), and they conformed to the National Institutes of Health guidelines on the ethical use of animals. For the lentiviral experiments, a bicistronic lentiviral vector system, pLox Syn-DsRed-Syn-GFP (pLox), was used by substituting either the DsRed or GFP coding sequence or both with the target cDNA sequence. For the Syt7 experiments, the cDNAs include mouse full-length or HA-tagged mouse Syt7 isoform α cDNA (Syt7^FL^; NM_018801.3), isoform 4 cDNA (Syt7^IF4^; NM_001373944.1), and isoform 4 cDNA carrying human *SYT7* L227M/Q247H (mouse L152M/Q172H) mutations (Syt7^IF4mut^). In the mouse Syt7 isoform α cDNA, Asp Ca^2+^ ligands 225, 227 (in C2A), 357 and 359 (in C2B) were mutated to Asn in Syt7^CLM^; cDNA for the C2AB domain (a.a. 134–403) was conjugated to the 1–20 amino acid sequence of GAP43 to generate Syt7^GAP43^. The lentiviral CRISPRi system was described previously [38]. The sgRNAs were designed to target the DNA region from −50 to 300 bp relative to the TSS of Syt1, Syt7, and Doc2a/b [39]. The sgRNA sequences are listed in **S1 Table**.

### Mouse primary neuronal culture

Mouse primary neuronal culture was performed as previously described [15]. Briefly, hippocampal neurons were dissected from newborn WT and homozygous Syt7 KO mice in the dark phase and incubated in 0.25% trypsin-EDTA (Life Technologies, Carlsbad, CA) for 15 min at 37°C. After washing with Hank’s Buffered Salt Solution plus 5 mM HEPES (Life Technologies), 20 mM D-glucose and 2% fetal bovine serum (FBS) (Gibco, Rockville, MD), the neurons were mechanically dissociated in culture medium and plated on poly-D-lysine-coated glass coverslips at a density of 50,000 to 100,000 cells/cm^2^. Cells were grown in Neurobasal-A medium (Life Technologies) supplemented with 2% B-27 (Life Technologies) and 2 mM glutamax (Life Technologies). Cultures were maintained at 37°C in a 5% CO_2_-humidified incubator.

### Lentivirus preparation and infection

Lentivirus preparation and infection was performed as previously described [15]. Briefly, lentiviral particles were generated by cotransfecting HEK 293FT cells with virus packaging vectors. HEK 293FT cells were maintained in Dulbecco’s modified eagle medium (DMEM) in 10% FBS, 100 units/ml streptomycin, and 100 mg/ml penicillin with 2 mM glutamax (Life Technologies). Transfection was performed using PEI (Polysciences, Warrington, PA). Five hours after transfection, the medium was changed. Virus supernatant was harvested 60 h posttransfection, filtered with a 0.22-μm PVDF filter (Millipore, Billerica, MA), ultracentrifuged at 25,000 rpm using a P28S rotor (Hitachi, Tokyo, Japan), and stocked in a final volume of 100 μl. The titer of the lentivirus used in all cell culture experiments was at least 5.0 × 10^8^ infectious units (IUs) per ml.

### Electrophysiology

Whole-cell recordings were performed in voltage-clamp mode using a MultiClamp 700B amplifier (Molecular Devices, Sunnyvale, CA). For acute slice preparation, P40–P60 animals were euthanized under Pentobarbitol *Sodium*. Brains were removed and placed in ice-cold solution containing (in mM) the following: 110 choline chloride, 25 NaHCO_3_, 7 MgCl_2_, 2.5 KCl, 1.3 NaH_2_PO_4_, 0.5 CaCl_2_, 1.3 Na-asorbate, 0.6 Na-pyruvate, and 20 D-glucose. On a Compresstome VF-330 vibrotome (Precisionary Instruments, Greenville, NC), 300-μm thick slices for hippocampus were prepared. Slices were transferred for 60 min to 33°C artificial cerebrospinal solution (ACSF) containing (in mM) the following: 124 NaCl, 26 NaHCO_3_, 10 glucose, 3 KCl, 2 CaCl_2_, 1.25 KH_2_PO_4_, and 1 MgCl_2_. Whole-cell patch-clamp recordings were performed from hippocampus slice at 33 ± 1°C with flow rates of 2 ml/min in ACSF containing 2 mM CaCl_2_ or SrCl_2_. For cultured neuron recording, the recording chamber was continuously perfused with a bath solution (128 mM NaCl, 30 mM glucose, 5 mM KCl, 1 mM MgCl_2_, 25 mM HEPES (pH 7.3)) containing 2 mM CaCl_2_ or SrCl_2_ via a Warner (Hamden, Connecticut) VC-6 drug delivery system. To record AMPAR-NMDAR-mEPSCs, 20 μM bicuculline (GABAR antagonist; Sigma, St. Louis, Missouri), 0.5 μM TTX (Tocris), 10 μM glycine (NMDAR co-agonist; Sigma), and 1 μM Strychnine (glycine receptor antagonist; Sigma) were applied, and MgCl_2_ was excluded from the extracellular solution. Depending on the experiments, 1 μM Ro25-6981 (Tocris, Bristol, UK) was used to abolish GluN2B activity, and 50 μM D-AP5 (Tocris) was applied to block all NMDARs. To record AMPAR-mEPSCs, glycine and Strychnine were removed, and AP5 and MgCl_2_ were applied. Patch pipettes were pulled from borosilicate glass and had resistances of 3 to 5 MΩ when filled with internal pipette solution (130 mM K-gluconate, 1 mM EGTA, 5 mM Na-phosphocreatine, 2 mM Mg-ATP, 0.3 mM Na-GTP, 5 mM QX-314, 10mM HEPES (pH 7.3)). The membrane potential was held at −70 mV. Data were acquired using pClamp10 software (Molecular Devices), sampled at 10 kHz, and filtered at 2 kHz. Offline data analysis of EPSCs was performed using Clampfit software (Molecular Devices).

### Differentiation of iPSC into DG-like neurons

The forebrain neural progenitor cells (NPCs) derived from BD patients and healthy people were characterized as previously described [27,40]. The information about the approving committee, informed consent, and clinical trial registration number of the iPSC studies was described in the original articles [27,40]. To obtain hippocampal DG-like neurons, NPCs were differentiated in DMEM/F12 supplemented with N2 (Life Technologies), B27 (Life Technologies), 20 ng/ml BDNF (Peprotech, Offenbach, Germany), 1 mM dibutyrl-cyclicAMP (Sigma), 200 nM ascorbic acid (Sigma), 1 μg/ml Laminin, and 620 ng/ml Wnt3a (R&D, Minneapolis, MN) for 3 to 4 weeks. Wnt3a was removed after 3 weeks. All cells used in the present study were verified as mycoplasma contamination free.

### Immunoblot analysis

Immunoblot analysis was performed as previously described [15]. Briefly, neurons were lysed in RIPA buffer (50 mM Tris-Cl (pH 8.0), 150 mM NaCl, 1% Nonidet P-40, 0.5% sodium deoxycholate, and 0.1% SDS) plus a complete protease inhibitor cocktail (Roche, Rotkreuz, Switzerland). Lysates were centrifuged and supernatants were subjected to SDS-PAGE. The blots were developed using an ECL kit (ThermoFisher, Waltham, USA). Protein levels were quantified by densitometry using NIH ImageJ 1.48 software. Primary antibodies were as follows: rabbit polyclonal anti-Syt1 antibody (1:2,000, Abcam, #ab131551), chicken polyclonal anti-HA antibody (1:1,000, Abcam, #ab9111), and mouse monoclonal anti-Actin antibody (1:5,000, Abcam, #ab6276).

### Immunoelectron microscopy

Cells were fixed in 4% paraformaldehyde (PFA) and 0.2% glutaraldehyde in phosphate buffered saline (PBS) at room temperature for 10 min. Cells were permeabilized in PBS containing 0.01% saponin for 5 min, blocked with PBS buffer containing 10% FBS, 10% goat serum, and 0.1% cold fish gelatin at room temperature for 40 min, and then exposed overnight to primary antibodies in blocking solution. The primary antibodies included rabbit polyclonal anti-HA antibody (1:200, Easybio, #BE2008). Samples were incubated with Flouronanogold anti-rabbit Fab’AleaxFlour 488 (1.4-nm diameter, Nanoprobes, #7204, 1:200) for 2 h and washed with PBS containing 0.1% cold fish gelatin for 10 min for 6 times. Then, the signal was intensified with a gold enhancement kit (GoldEnhance EM, Nanoprobes, #2114) at room temperature for 2 min. The samples were postfixed in 1% OsO4 containing 1.5% potassium ferrocyanide, then dehydrated in a series of graded ethanol solutions and embedded in epoxy resin. Ultrathin sections were collected and stained with uranyl acetate and lead citrate and observed and analyzed using a Hitachi H-7650B transmission electron microscope.

### Reverse transcription PCR

Total RNA was isolated using Trizol (Life Technologies) according to the manufacturer’s instructions. cDNA was synthesized using SuperScript III Reverse Transcription Kit (Life Technologies). The primer sequences for PCR are listed in **S2 Table**. qRT-PCR was performed on a Bio-Rad CFX96 thermal cycler using SYBR green supermix (Bio-Rad, Hercules, CA) and gene-specific primers. Quantitative analysis was performed employing the ΔΔCT method and the GAPDH as the endogenous control.

### Duolink in situ proximity ligation assay (PLA)

The Duolink in situ PLA was performed using a kit purchased from Sigma (Cat# DUO92008, 92004 and 92002) according to the manufacturer’s instructions. Briefly, neurons were fixed for 10 min in 4% PFA in PBS, followed by 10 min in permeabilization buffer containing 0.1% Triton X-100. After blocking for 1 h in PBS plus 0.1% Triton X-100 and 1% BSA, cells were incubated for 1 h with primary antibodies and washed 3 times with PBS. The PLA plus and minus probes were added, and the cells were incubated for 1 h at 37°C. After that, ligation and amplification were performed. Cells were stained with DAPI (0.1 μg/ml, Sigma) in PBS for 10 min at room temperature. All images were collected using an Olympus FV1200 Confocal microscope (Olympus, Japan) with a 60X objective (NA 1.40). Images were acquired using Fluoview software version 1.6 (Olympus) and analyzed using ImageJ software (NIH). The primary antibodies included rabbit polyclonal anti-Stx1a antibody (1:500, Abcam, #ab41453), mouse monoclonal anti-SNAP25 antibody (1:500, Synaptic Systems, #111011), and rabbit polyclonal anti-Syt7 antibody (1:200, Synaptic Systems, #105173).

### STORM imaging

STORM imaging analysis was performed as previously described [15]. Briefly, cultured hippocampal neurons were fixed with 4% PFA and 0.1% glutaradehyde in PBS (pH 7.4) for 10 min, followed by washing off excess PFA and reducing unreacted aldehyde groups with 0.1% sodium borohydride (NaBH_4_) in PBS. Cells were then blocked and permeabilized in blocking buffer (3% w/v BSA, 0.2% v/v Triton X-100 in PBS) for 1 h at room temperature, followed by incubation with primary and secondary antibodies, each for 2 h at room temperature. After washing, cells were postfixed for 10 min with 4% PFA and 0.1% glutaraldehyde in PBS and used for STORM imaging. Primary antibodies include rabbit polyclonal anti-Syt7 antibody (1:200, Synaptic Systems, #105173), mouse monoclonal anti-PSD95 antibody (1:300, Millipore, #MAB1598), and Guinea pig polyclonal anti-vGLUT1 antibody (1:200, Synaptic Systems, #135304). Secondary antibodies include donkey anti-rabbit AleaxFlour568 antibody (1:500, Life Technologies, #a10042), goat anti-mouse ATTO488 antibody (1:500, Lockland, #610-152-121S), and goat anti-Guinea pig AleaxFlour568 antibody (1:500, Life Technologies, #a11075). Imaging experiments were performed using a Nikon combined Confocal A1/SIM/STORM system with an Andor EMCCD camera iXON 897. STORM imaging was performed in imaging buffer containing 50 mM Tris (pH 8.0) and 10 mM NaCl, an oxygen scavenging system consisting of 1.2 mg/ml glucose oxidase (Sigma-Aldrich), 73 μm/ml catalase (Sigma-Aldrich) and 10% (w/v) glucose, and 304 mM β-mercaptoethylamine (Sigma-Aldrich). Data analysis was performed using NIS-Elements AR (Nikon, Tokyo, Japan) software.

### Statistical analysis

Data are shown as mean ± SEM of the values from at least 3 independent experiments. The numerical data are included in S1 Data. For experiments in **Fig 2D–2I**, statistical significance was evaluated using two-tailed paired Student *t* test at *P* < 0.05. For experiments in **Fig 3**, ANOVA was used. For all other experiments, statistical significance was evaluated using two-tailed unpaired Student *t* test at *P* < 0.05.

## Supporting information

S1 Fig Analysis of Sr^2+^-triggered mEPSCs in Syt1 KO or Doc2 KD hippocampal neurons. (A) Representative traces of Ca^2+^- and Sr^2+^-triggered mEPSCs recorded from cultured hippocampal neurons with Syt1 KO or Doc2a/2b KD. (B) Analysis of mEPSC frequency normalized to the Ca^2+^ group. WT, *n =* 16 (Ca^2+^)/20 (Sr^2+^); Syt1 KO, *n* = 12/12; Doc2a/2b KD, *n* = 10/15. Student *t* test; *P < 0.05; **P < 0.001; error bars, SEM. The numerical data underlying this figure are included in S1 Data. mEPSC, miniature excitatory postsynaptic current; KD, knockdown; KO, knockout; Syt1, synaptotagmin-1; WT, wild-type.
(TIF)

S2 Fig Syt7 triggers spontaneous neurotransmitter release in the absence of Syt1. (A) Representative traces of mEPSCs recorded from cultured Syt1 KD hippocampal neurons. (B) Quantitative analysis of the frequency (left) and amplitude (right) of Ca^2+^- and Sr^2+^-triggered mEPSCs in Syt1 KD neurons. *n* = 16. (C) Sample traces of mEPSCs recorded from cultured Syt1/Syt7 dKD hippocampal neurons. (D) Bar graphs showing the frequency (left) and amplitude (right) of Ca^2+^- and Sr^2+^-triggered mEPSCs in Syt1/Syt7 dKD neurons. *n =* 16. Student *t* test. *P < 0.05; error bars, SEM. The numerical data underlying this figure are included in S1 Data. dKD, double KD; mEPSC, miniature excitatory postsynaptic current; KD, knockdown; Syt1, synaptotagmin-1; Syt7, synaptotagmin-7.
(TIF)

S3 Fig Syt7 triggered spontaneous GluN2B-NMDAR activity in the absence of Syt1. (A) Average traces of AMPAR/NMDAR-mEPSCs recorded in cultured Syt1 KD (upper) or Syt1/Syt7 dKD (lower) hippocampal neurons. (B, C) Bar graphs summarizing the decay time (B) and total charge (C) of Syt1 KD (left) or Syt1/Syt7 dKD (right) neurons. *n* = 16 for all groups. Student *t* test; **P < 0.001; error bars, SEM. The numerical data underlying this figure are included in S1 Data. AMPAR, AMPA receptor; dKD, double KD; KD, knockdown; mEPSC, miniature excitatory postsynaptic current; Syt1, synaptotagmin-1; Syt7, synaptotagmin-7.
(TIF)

S4 Fig Expression and localization of GluN2B-NMDARs in Syt7 KO hippocampal neurons. (A) Immunoblots of GluN2B in WT and Syt7 KO hippocampal tissues (left) and qRT-PCR analysis of GluN2B in cultured WT and Syt7 KO neurons. *n =* 3. (B) Immunoblots of GluN2A and GluN2B in the hippocampus of postnatal days 0, 7, and 35 (P0/P7/P35) WT and Syt7 KO mice. (C) Sample STORM images showing localization of GluN2B in the synapses of cultured WT (upper) and Syt7 KO (lower) hippocampal neurons. From left to right, the 2D view, the side view, and the face view of sample synapses. Antibodies specific for vGLUT1 and PSD95 were employed to delineate the presynaptic boutons and PSD, respectively. Scale bar, 100 nm. Scale bar, 100 nm. (D) Shortest distance of the GluN2B signal to the PSD center in the WT and Syt7 KO neurons. Curves are fitted Gaussian curve. *n =* 50. Student *t* test; *P < 0.05; error bars, SEM. The numerical data underlying this figure are included in S1 Data. KO, knockout; qRT-PCR, quantitative reverse transcription PCR; PSD, postsynaptic density; STORM, stochastic optical reconstruction microscopy; Syt7, synaptotagmin-7; WT, wild-type.
(TIF)

S5 Fig Duolink analysis of co-localization of Syt7 and Syt7^GAP43^ with SNARE proteins in cultured Syt7 KO neurons. (A) Schematic rationale for the rationale of Duolink PLA. The 2 probes can generate PLA fluorescence signal when they are within a 40-nm distance. (B) Sample fluorescence images showing the Duolink puncta of SNAP-25 with ligation to Stx1a (upper) or Syt7 (lower). (C) Bar graphs showing the puncta area (upper) and density (lower) of SNAP-25 with ligation to Stx1a (*n* = 10) or Syt7 (*n* = 12). (D) Sample fluorescence images showing the Duolink puncta of Stx1a/Syb2 with ligation to Syt7GAP43-HA or Syt7FL-HA in Syt7 KO neurons. (E) Bar graphs showing the puncta area (upper) and density (lower) of Stx1a/Syb2 with ligation to Syt7GAP43-HA (*n =* 7) or Syt7FL-HA (*n* = 8) in Syt7 KO neurons. Student *t* test; *P < 0.05; **P < 0.001; error bars, SEM. The numerical data underlying this figure are included in S1 Data. HRP, horseradish peroxidase; KO, knockout; PLA, proximity ligation assay; Stx1a, syntaxin-1A; Syb2, synaptobrevin-2; Syt7, synaptotagmin-7.
(TIF)

S6 Fig Analysis of mEPSCs recorded in cultured Syt7 KO hippocampal neurons expressing Syt7^GAP43^. (A–D) ANOVA analysis of results in Fig 3E(A), 3F(B), 3H(C), and 3I(D). (E, F) Bar graphs showing the frequency (E) and amplitude (F) of AMPAR/NMDAR-mEPSCs in Syt7^GAP43^-expressing neurons following GluN2B blockade. WT, *n* = 12/9/7; KO, *n* = 16/13/8; Syt7^FL^, *n* = 12/12/8; Syt7^GAP43^, *n* = 14/13/11. (A–D) ANOVA; (E, F) Student *t* test; *P < 0.05; **P < 0.001; error bars, SEM. The numerical data underlying this figure are included in S1 Data. AMPAR, AMPA receptor; FL, full-length; GAP, GAP43; KO, knockout; mEPSC, miniature excitatory postsynaptic current; Syt7, synaptotagmin-7; WT, wild-type.
(TIF)

S7 Fig Spontaneous GluN2B activity in BD patient iPSC-derived hippocampal neurons. (A) Sample immunostaining images showing the expression of Prox1 in the iPSC-derived neurons. Scale bar, 40 μm. (B) Sample traces showing evoked APs in the Prox1^+^ neurons. (C–F) Bar graphs summarizing the effects of Ro25-6981 on the decay time of AMPAR/NMDAR-mEPSCs in the HC neurons (C), BD-I neurons (D), and BD-I neurons overexpressing Syt7^FL^ (E) or Syt7^IF4mut^ (F) derived from 3 iPSC lines. (G–J) Bar graphs summarizing the charge transfer of AMPAR/NMDAR-mEPSCs in the HC neurons (G), BD-I neurons (H), and BD-I neurons overexpressing Syt7^FL^ (I) or Syt7^IF4mut^ (J) derived from 3 iPSC lines. For all groups, *n =* 6–12 neurons per cell line. Student *t* test; *P < 0.05; **P < 0.001; error bars, SEM. The numerical data underlying this figure are included in S1 Data. AMPAR, AMPA receptor; AP, action potential; BD, bipolar disorder; HC, healthy control; iPSC, induced pluripotent stem cell; mEPSC, miniature excitatory postsynaptic current.
(TIF)

S8 Fig Analyses of synapse density and RRP in cultured hippocampal neurons expressing Syt7 mutants. (A) Representative immunostaining images showing the expression of vGLUT1 and MAP2 in cultured hippocampal neurons. Scale bar, 50 μm. (B) Sample immunostaining images showing the vGLUT1 puncta along the MAP2-expression dendrites in the WT and Syt7 KO neurons. Scale bar, 4 μm. (C) Quantitative analysis of vGLUT1 puncta density in Syt7 KO and mutant-expressing neurons. (D, E) Representative traces (D) and cumulative charge transfer (E) of EPSCs evoked by a 2-s 10-Hz train stimulation in WT neurons, Syt7 KO neurons, and KO neurons expressing Syt^FL^, Syt7^CLM^, Syt7^IF4mut^, or Syt7^GAP43^. (F) Summary of the RRP size defined by y-intercepts of linear function fitted to the last 3–5 data points of the EPSC trains. WT, *n* = 17; KO, *n* = 12; Syt^FL^, *n* = 9; Syt^CLM^, *n* = 11; Syt^IF4mut^, *n* = 15; Syt^GAP43^, *n* = 13. (G) Sample immunostaining images showing the co-localization of Syt7 ^IF4mut^ and presynaptic vGLUT1 puncta. Student *t* test; **P < 0.001; error bars, SEM. The numerical data underlying this figure are included in S1 Data. CLM, calcium ligand mutant; EPSC, excitatory postsynaptic current; FL, full-length; KO, knockout; RRP, readily releasable SV pool; Syt7, synaptotagmin-7; WT, wild-type.
(TIF)

S1 Table Oligonucleotides used for CRISPR/Cas9-mediated gene deletion through embryo injection. (DOCX)

S2 Table Sequence of primers for qRT-PCR analysis. (DOCX)

S1 Data Numerical data underlying Figs 1–4 and S1–S8. Excel spreadsheet containing in separate sheets the numerical data underlying Figs 1–4 and S1–S8, respectively.
(XLSX)

S1 Raw images Uncropped western blots data related to Figs 3B and S4A and S4B. (PDF)

## Abbreviations

ACSF: artificial cerebrospinal solution
AMPAR: AMPA receptor
AP: action potential
AZ: active zone
BD: bipolar disorder
CRISPRi: CRISPR interference
DG: dentate gyrus
dKD: double KD
DMEM: Dulbecco’s modified eagle medium
FBS: fetal bovine serum
immuno-EM: immunoelectron microscopy
iPSC: induced pluripotent stem cell
IU: infectious unit
KD: knockdown
KO: knockout
LTP: long-term potentiation
mEPSC: miniature excitatory postsynaptic current
NPC: neural progenitor cell
PBS: phosphate buffered saline
PFA: paraformaldehyde
PLA: proximity ligation assay
PSD: postsynaptic density
qKD: quadra KD
qRT-PCR: quantitative reverse transcription PCR
RRP: readily releasable SV pool
SNP: single nucleotide polymorphism
Stx1a: syntaxin-1A
STORM: stochastic optical reconstruction microscopy
SV: synaptic vesicle
Syb2: synaptobrevin-2
Syt7: synaptotagmin-7
tKD: triple KD
WT: wild-type
