# Supplementary figures and images for: Synaptotagmin-7–mediated activation of spontaneous NMDAR currents is disrupted in bipolar disorder susceptibility variants
Source: PLoS Biol. 2021 Jul 6;19(7):e3001323. doi: 10.1371/journal.pbio.3001323 (PMC8284830; doi:10.1371/journal.pbio.3001323)

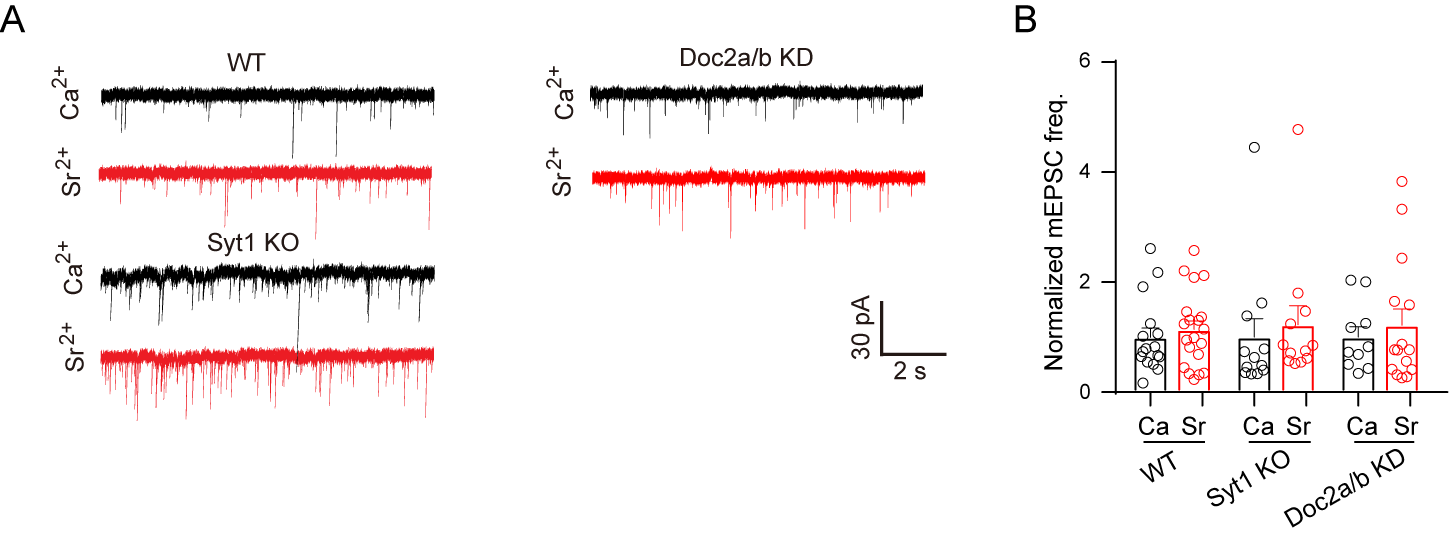

Supplement: S1 Fig — (A) Representative traces of Ca2+- and Sr2+-triggered mEPSCs recorded from cultured hippocampal neurons with Syt1 KO or Doc2a/2b KD. (B) Analysis of mEPSC frequency normalized to the Ca2+ group. WT, n = 16 (Ca2+)/20 (Sr2+); Syt1 KO, n = 12/12; Doc2a/2b KD, n = 10/15. Student t test; *P < 0.05; **P < 0.001; error bars, SEM. The numerical data underlying this figure are included in S1 Data. mEPSC, miniature excitatory postsynaptic current; KD, knockdown; KO, knockout; Syt1, synaptotagmin-1; WT, wild-type. (TIF) [file pbio.3001323.s001.tif]

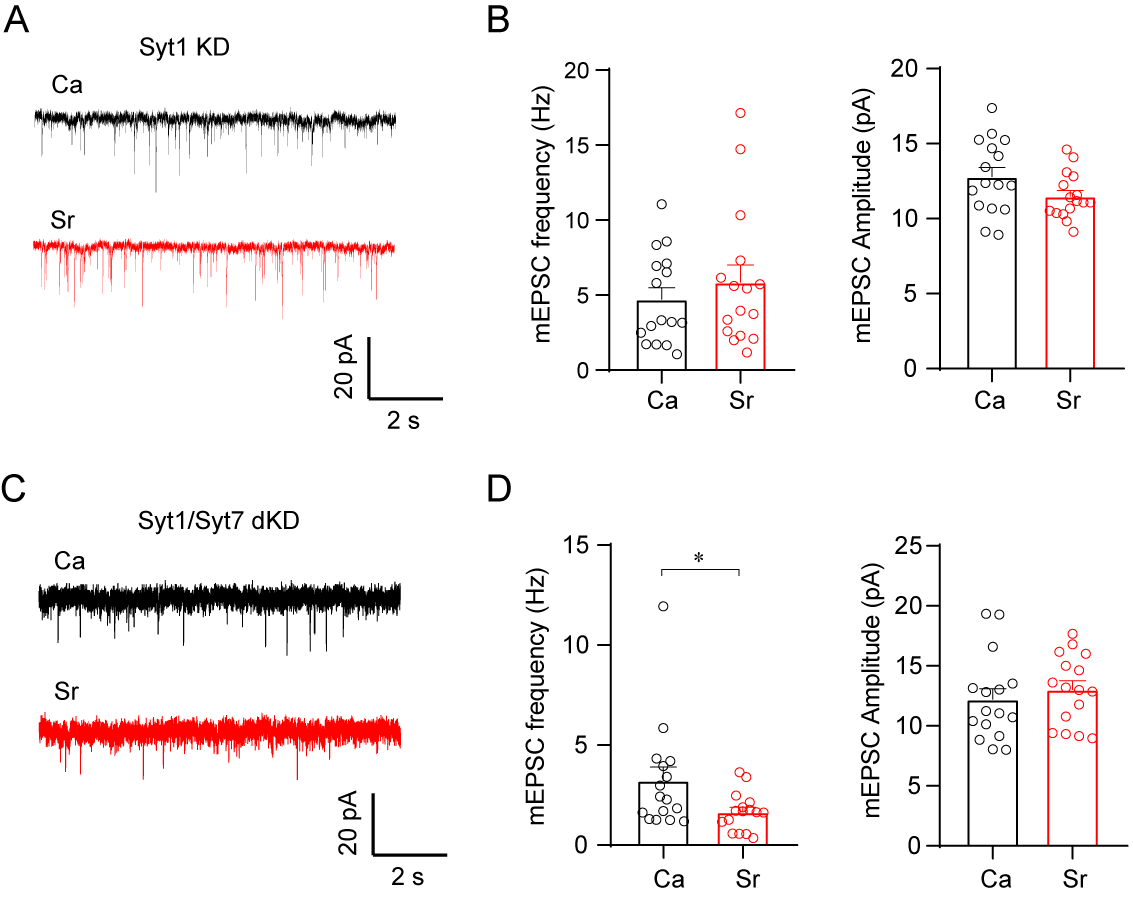

Supplement: S2 Fig — (A) Representative traces of mEPSCs recorded from cultured Syt1 KD hippocampal neurons. (B) Quantitative analysis of the frequency (left) and amplitude (right) of Ca2+- and Sr2+-triggered mEPSCs in Syt1 KD neurons. n = 16. (C) Sample traces of mEPSCs recorded from cultured Syt1/Syt7 dKD hippocampal neurons. (D) Bar graphs showing the frequency (left) and amplitude (right) of Ca2+- and Sr2+-triggered mEPSCs in Syt1/Syt7 dKD neurons. n = 16. Student t test. *P < 0.05; error bars, SEM. The numerical data underlying this figure are included in S1 Data. dKD, double KD; mEPSC, miniature excitatory postsynaptic current; KD, knockdown; Syt1, synaptotagmin-1; Syt7, synaptotagmin-7. (TIF) [file pbio.3001323.s002.tif]

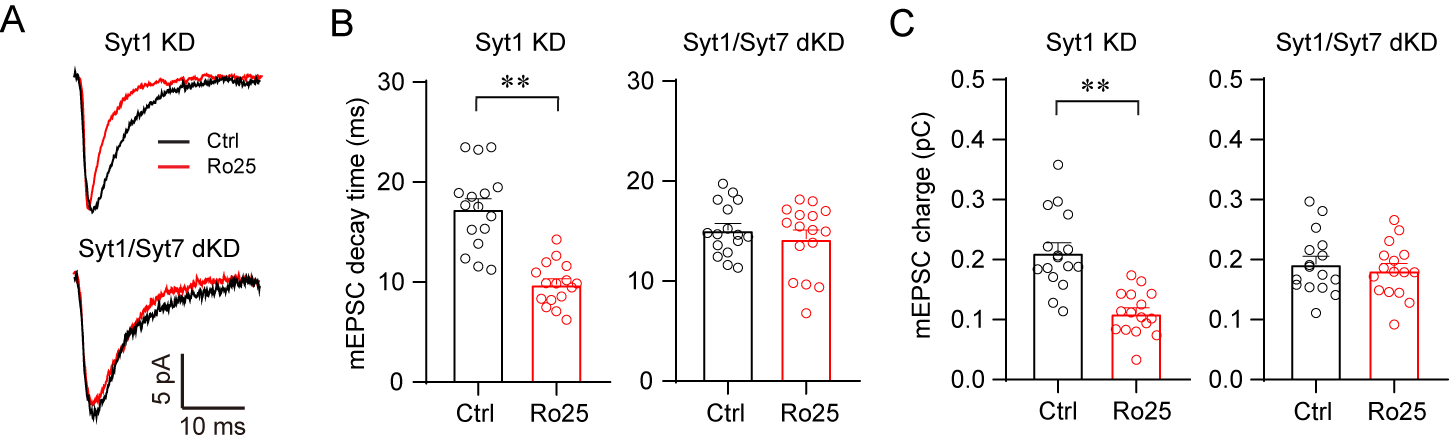

Supplement: S3 Fig — (A) Average traces of AMPAR/NMDAR-mEPSCs recorded in cultured Syt1 KD (upper) or Syt1/Syt7 dKD (lower) hippocampal neurons. (B, C) Bar graphs summarizing the decay time (B) and total charge (C) of Syt1 KD (left) or Syt1/Syt7 dKD (right) neurons. n = 16 for all groups. Student t test; **P < 0.001; error bars, SEM. The numerical data underlying this figure are included in S1 Data. AMPAR, AMPA receptor; dKD, double KD; KD, knockdown; mEPSC, miniature excitatory postsynaptic current; Syt1, synaptotagmin-1; Syt7, synaptotagmin-7. (TIF) [file pbio.3001323.s003.tif]

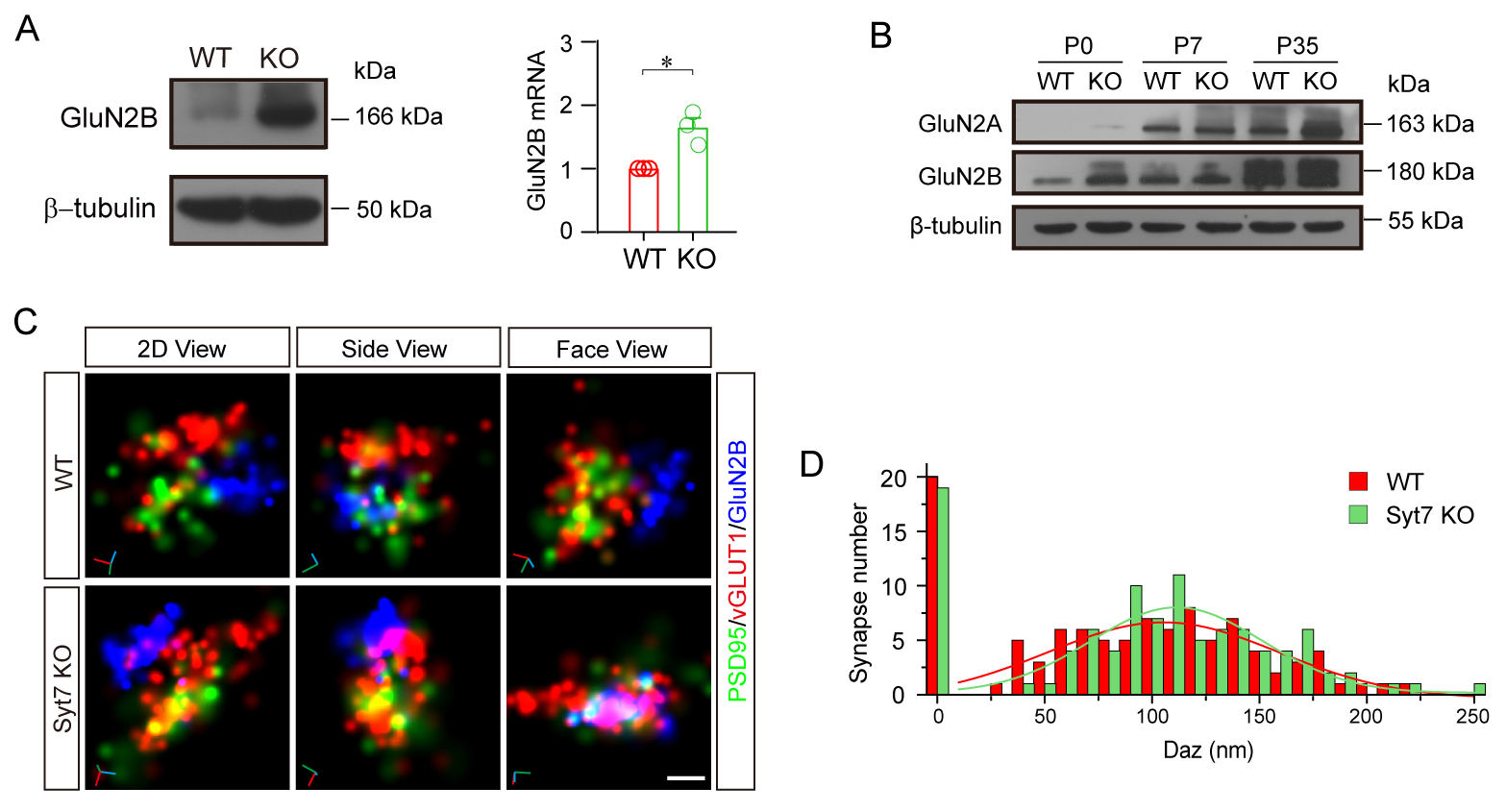

Supplement: S4 Fig — (A) Immunoblots of GluN2B in WT and Syt7 KO hippocampal tissues (left) and qRT-PCR analysis of GluN2B in cultured WT and Syt7 KO neurons. n = 3. (B) Immunoblots of GluN2A and GluN2B in the hippocampus of postnatal days 0, 7, and 35 (P0/P7/P35) WT and Syt7 KO mice. (C) Sample STORM images showing localization of GluN2B in the synapses of cultured WT (upper) and Syt7 KO (lower) hippocampal neurons. From left to right, the 2D view, the side view, and the face view of sample synapses. Antibodies specific for vGLUT1 and PSD95 were employed to delineate the presynaptic boutons and PSD, respectively. Scale bar, 100 nm. Scale bar, 100 nm. (D) Shortest distance of the GluN2B signal to the PSD center in the WT and Syt7 KO neurons. Curves are fitted Gaussian curve. n = 50. Student t test; *P < 0.05; error bars, SEM. The numerical data underlying this figure are included in S1 Data. KO, knockout; qRT-PCR, quantitative reverse transcription PCR; PSD, postsynaptic density; STORM, stochastic optical reconstruction microscopy; Syt7, synaptotagmin-7; WT, wild-type. (TIF) [file pbio.3001323.s004.tif]

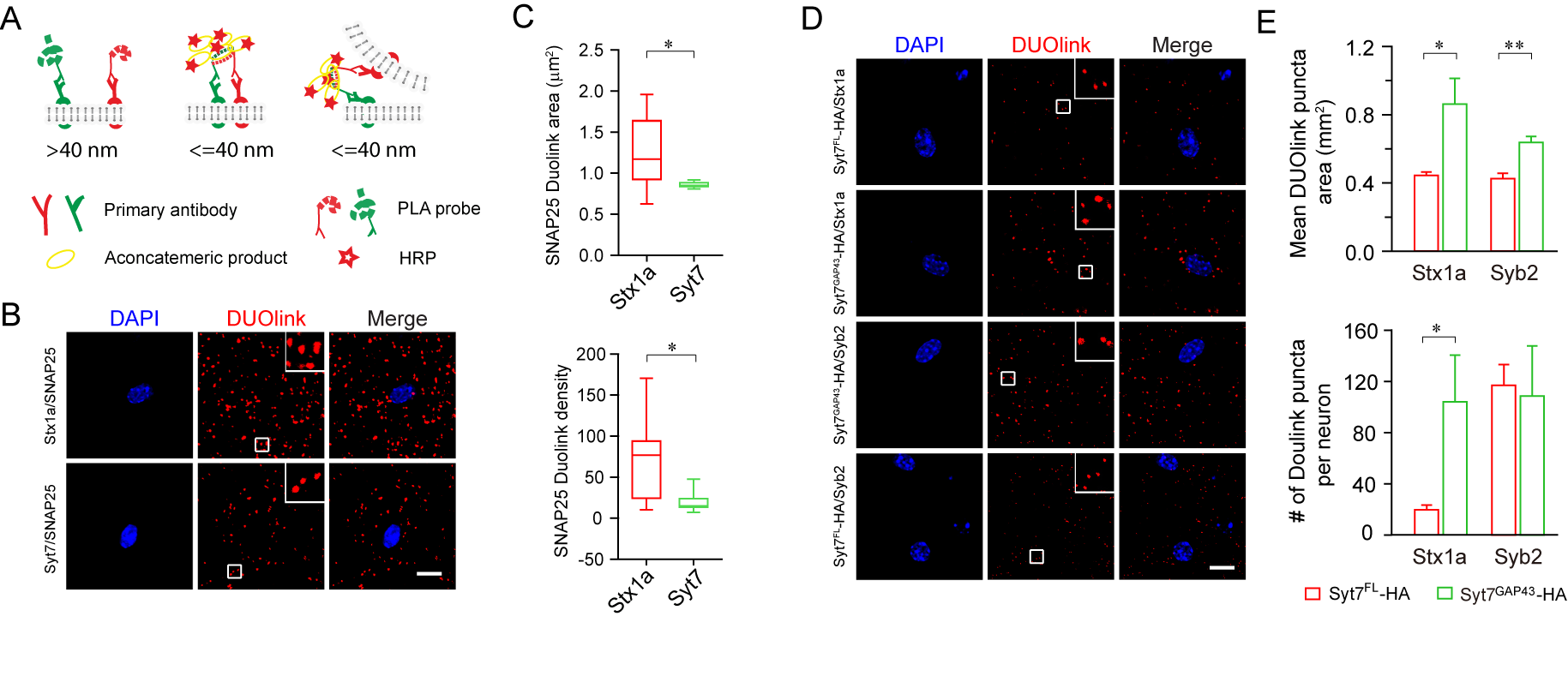

Supplement: S5 Fig — (A) Schematic rationale for the rationale of Duolink PLA. The 2 probes can generate PLA fluorescence signal when they are within a 40-nm distance. (B) Sample fluorescence images showing the Duolink puncta of SNAP-25 with ligation to Stx1a (upper) or Syt7 (lower). (C) Bar graphs showing the puncta area (upper) and density (lower) of SNAP-25 with ligation to Stx1a (n = 10) or Syt7 (n = 12). (D) Sample fluorescence images showing the Duolink puncta of Stx1a/Syb2 with ligation to Syt7GAP43-HA or Syt7FL-HA in Syt7 KO neurons. (E) Bar graphs showing the puncta area (upper) and density (lower) of Stx1a/Syb2 with ligation to Syt7GAP43-HA (n = 7) or Syt7FL-HA (n = 8) in Syt7 KO neurons. Student t test; *P < 0.05; **P < 0.001; error bars, SEM. The numerical data underlying this figure are included in S1 Data. HRP, horseradish peroxidase; KO, knockout; PLA, proximity ligation assay; Stx1a, syntaxin-1A; Syb2, synaptobrevin-2; Syt7, synaptotagmin-7. (TIF) [file pbio.3001323.s005.tif]

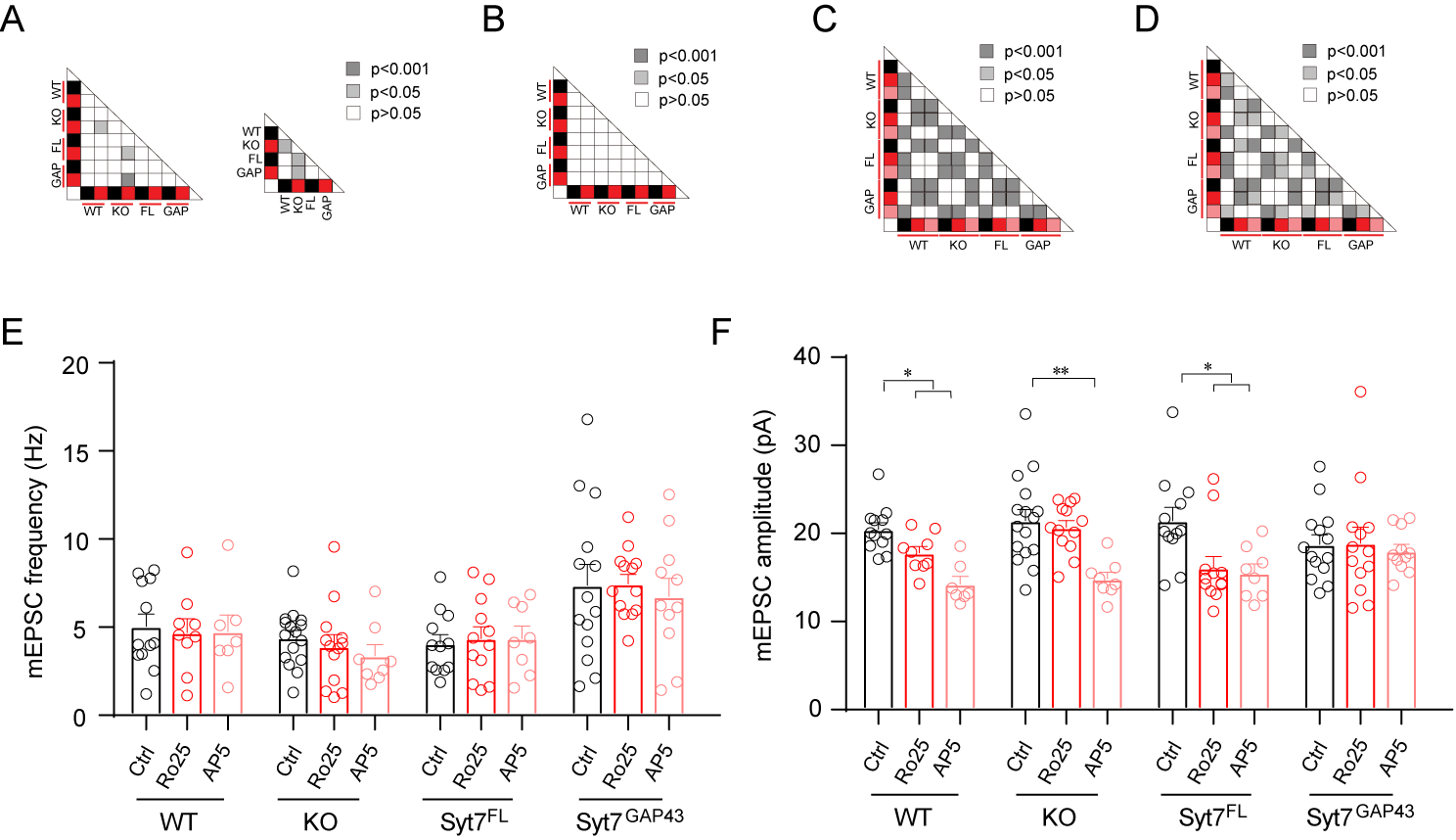

Supplement: S6 Fig — (A–D) ANOVA analysis of results in Fig 3E(A), 3F(B), 3H(C), and 3I(D). (E, F) Bar graphs showing the frequency (E) and amplitude (F) of AMPAR/NMDAR-mEPSCs in Syt7GAP43-expressing neurons following GluN2B blockade. WT, n = 12/9/7; KO, n = 16/13/8; Syt7FL, n = 12/12/8; Syt7GAP43, n = 14/13/11. (A–D) ANOVA; (E, F) Student t test; *P < 0.05; **P < 0.001; error bars, SEM. The numerical data underlying this figure are included in S1 Data. AMPAR, AMPA receptor; FL, full-length; GAP, GAP43; KO, knockout; mEPSC, miniature excitatory postsynaptic current; Syt7, synaptotagmin-7; WT, wild-type. (TIF) [file pbio.3001323.s006.tif]

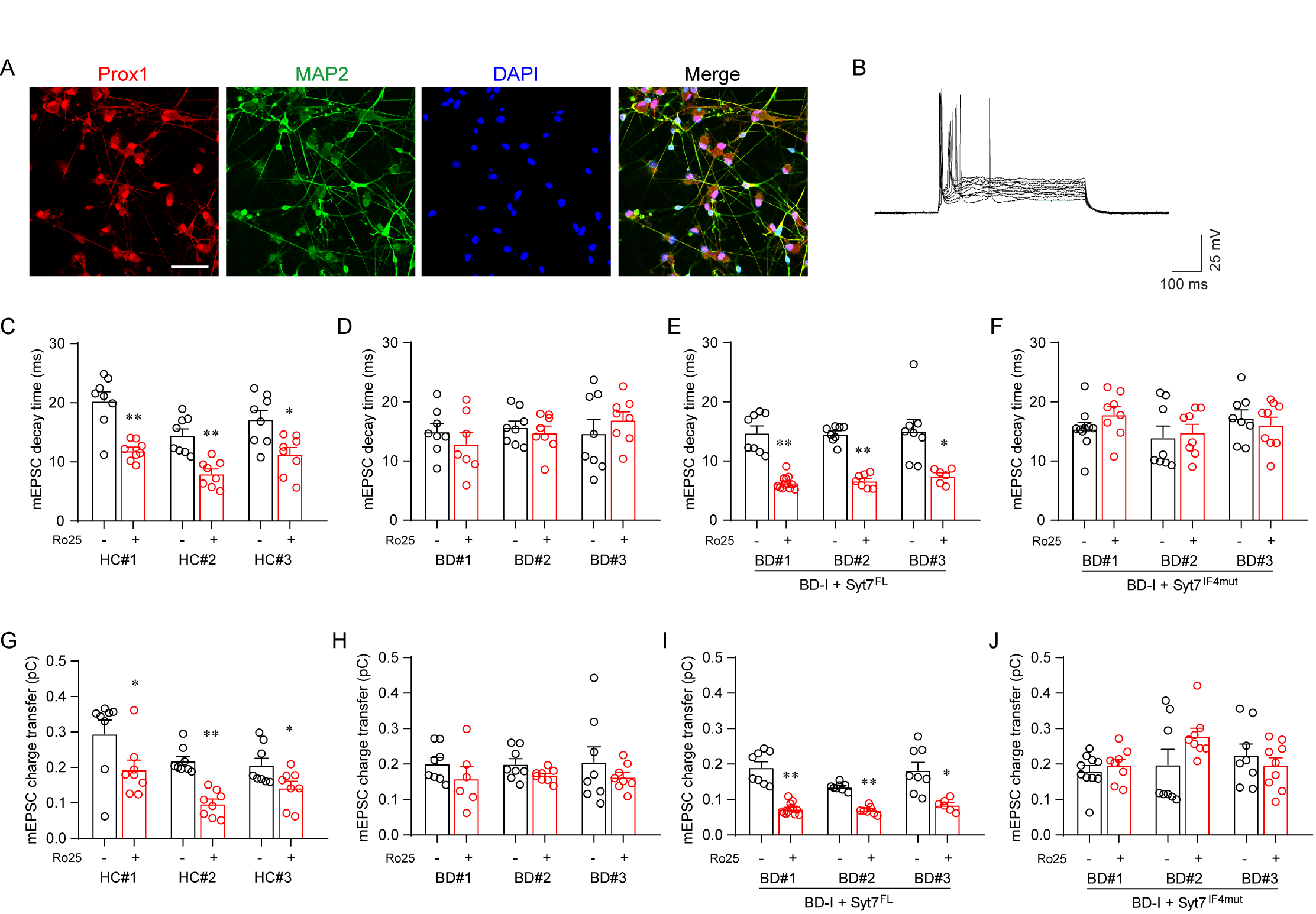

Supplement: S7 Fig — (A) Sample immunostaining images showing the expression of Prox1 in the iPSC-derived neurons. Scale bar, 40 μm. (B) Sample traces showing evoked APs in the Prox1+ neurons. (C–F) Bar graphs summarizing the effects of Ro25-6981 on the decay time of AMPAR/NMDAR-mEPSCs in the HC neurons (C), BD-I neurons (D), and BD-I neurons overexpressing Syt7FL (E) or Syt7IF4mut (F) derived from 3 iPSC lines. (G–J) Bar graphs summarizing the charge transfer of AMPAR/NMDAR-mEPSCs in the HC neurons (G), BD-I neurons (H), and BD-I neurons overexpressing Syt7FL (I) or Syt7IF4mut (J) derived from 3 iPSC lines. For all groups, n = 6–12 neurons per cell line. Student t test; *P < 0.05; **P < 0.001; error bars, SEM. The numerical data underlying this figure are included in S1 Data. AMPAR, AMPA receptor; AP, action potential; BD, bipolar disorder; HC, healthy control; iPSC, induced pluripotent stem cell; mEPSC, miniature excitatory postsynaptic current. (TIF) [file pbio.3001323.s007.tif]

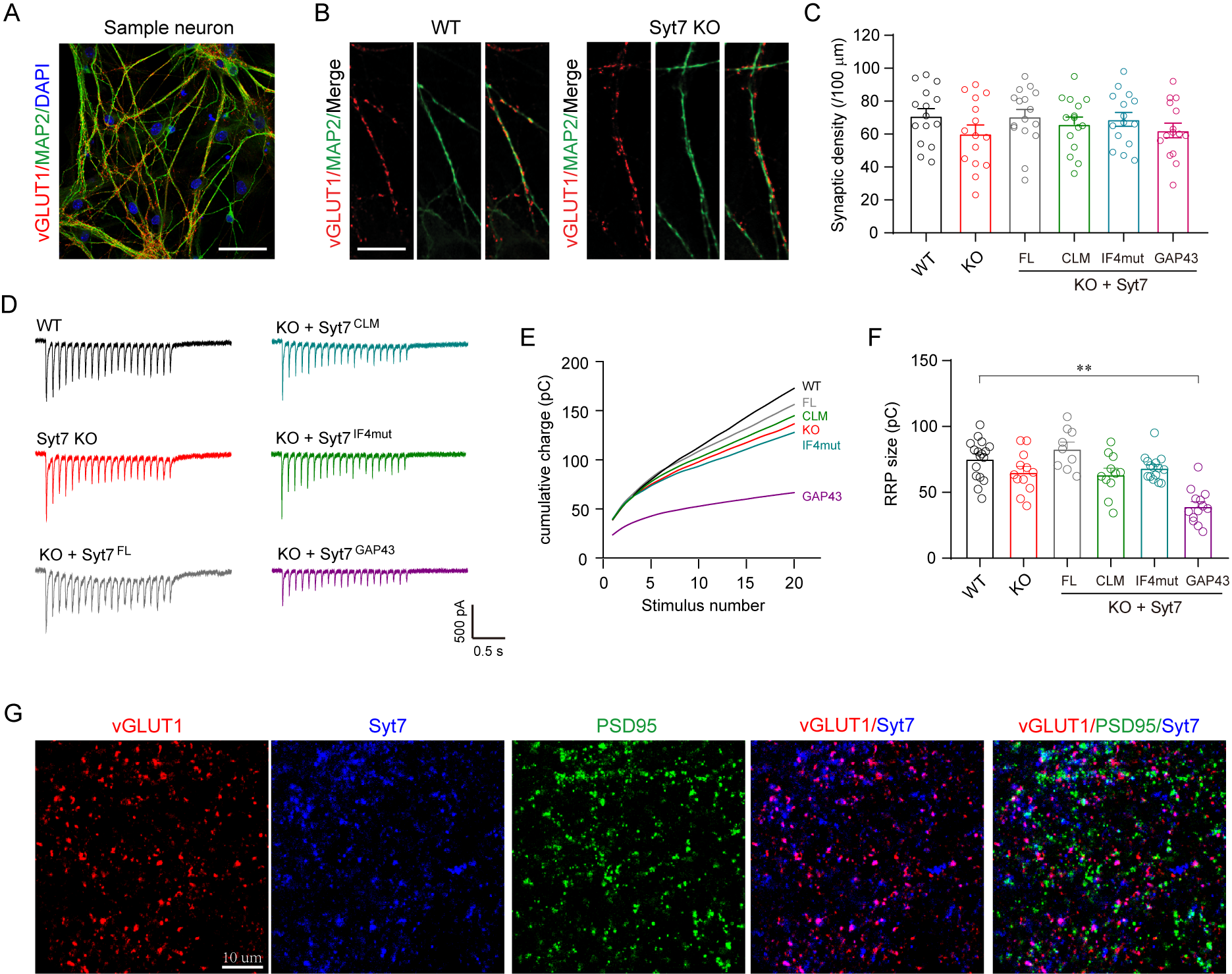

Supplement: S8 Fig — (A) Representative immunostaining images showing the expression of vGLUT1 and MAP2 in cultured hippocampal neurons. Scale bar, 50 μm. (B) Sample immunostaining images showing the vGLUT1 puncta along the MAP2-expression dendrites in the WT and Syt7 KO neurons. Scale bar, 4 μm. (C) Quantitative analysis of vGLUT1 puncta density in Syt7 KO and mutant-expressing neurons. (D, E) Representative traces (D) and cumulative charge transfer (E) of EPSCs evoked by a 2-s 10-Hz train stimulation in WT neurons, Syt7 KO neurons, and KO neurons expressing SytFL, Syt7CLM, Syt7IF4mut, or Syt7GAP43. (F) Summary of the RRP size defined by y-intercepts of linear function fitted to the last 3–5 data points of the EPSC trains. WT, n = 17; KO, n = 12; SytFL, n = 9; SytCLM, n = 11; SytIF4mut, n = 15; SytGAP43, n = 13. (G) Sample immunostaining images showing the co-localization of Syt7 IF4mut and presynaptic vGLUT1 puncta. Student t test; **P < 0.001; error bars, SEM. The numerical data underlying this figure are included in S1 Data. CLM, calcium ligand mutant; EPSC, excitatory postsynaptic current; FL, full-length; KO, knockout; RRP, readily releasable SV pool; Syt7, synaptotagmin-7; WT, wild-type. (TIF) [file pbio.3001323.s008.tif]

Fig 3B

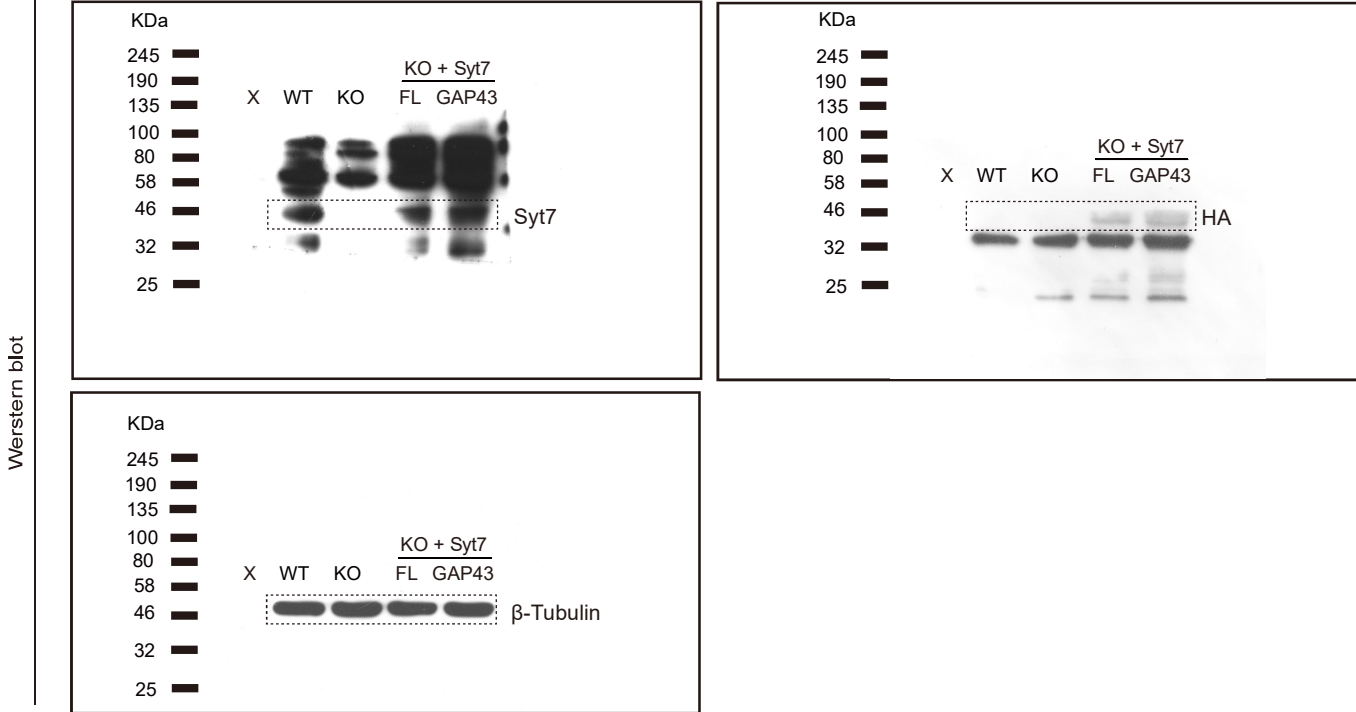

S4A Fig

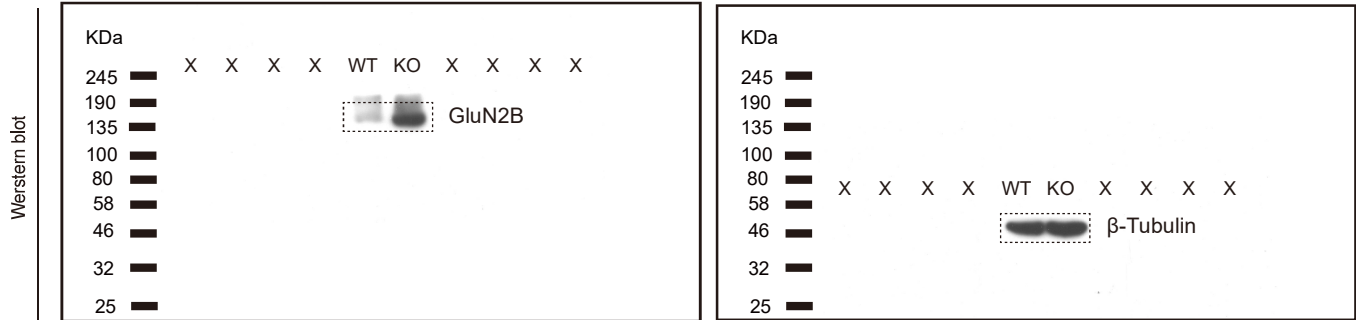

S4B Fig

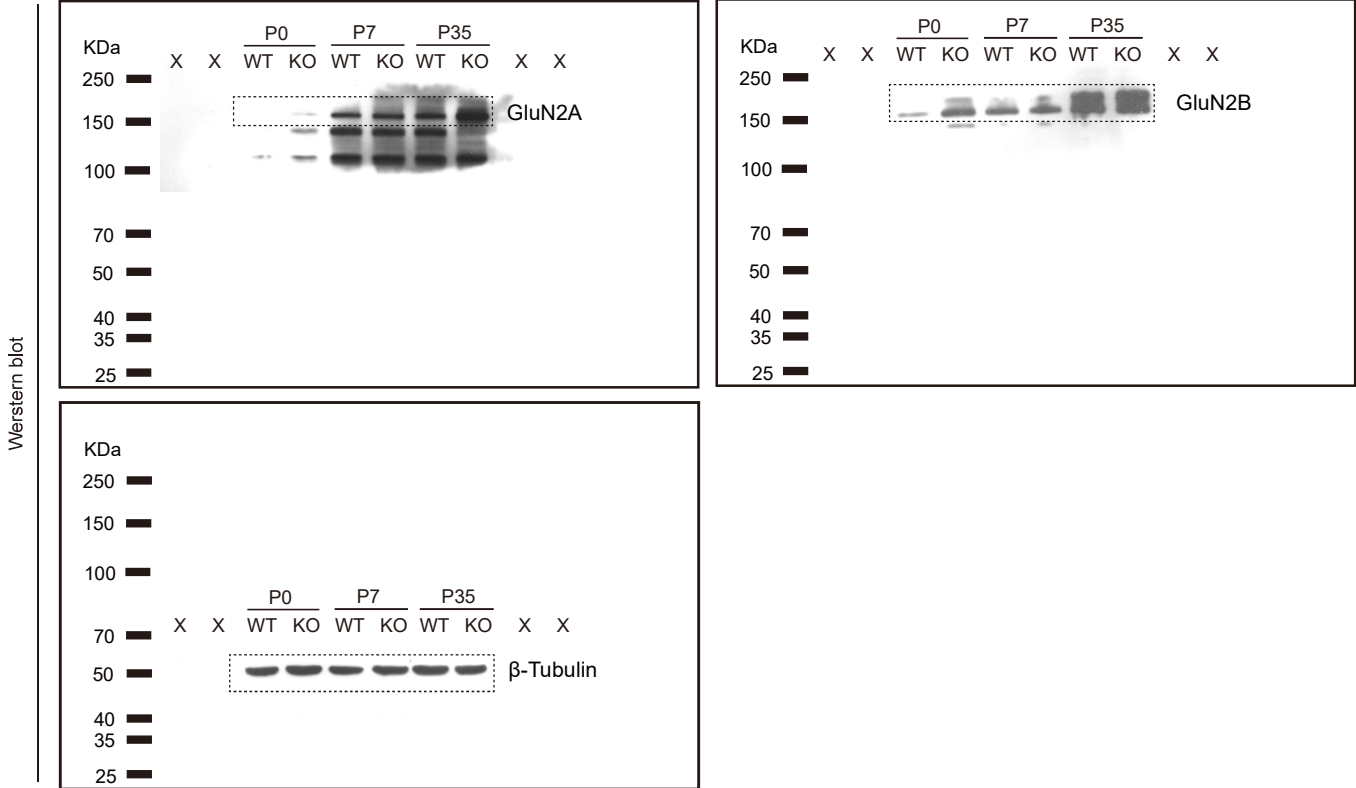

Supplement: S1 Raw images — (PDF) [file pbio.3001323.s012.pdf]
