# Supplementary material for: Synaptotagmin-7–mediated activation of spontaneous NMDAR currents is disrupted in bipolar disorder susceptibility variants
Source: PLoS Biol. 2021 Jul 6;19(7):e3001323. doi: 10.1371/journal.pbio.3001323 (PMC8284830; doi:10.1371/journal.pbio.3001323)
Supplement: S1 Table — (DOCX) [file pbio.3001323.s009.docx]

**S1 Table. Oligonucleotides used for CRISPR/Cas9-mediated gene deletion through embryo injection**

| Gene | SgRNA Sequence (5' to 3') |
| --- | --- |
| *Syt1* | 5’-TGTAACCGGGGCAAGCCCCC-3’ |
| *Doc2a* | 5’-ACACCGGGCGCCTCTCGCGG-3’ |
| *Doc2b* | 5’-GCCGCGTCAGGGCCGTCCGG-3’ |
| *Syt7* | 5’-TGCTCCGCGGGCGGCCCGGA-3’ |
| *Scramble* | 5’-GCGCCAAACGTGCCCTGACG-3’ |
