# Supplementary material for: Synaptotagmin-7–mediated activation of spontaneous NMDAR currents is disrupted in bipolar disorder susceptibility variants
Source: PLoS Biol. 2021 Jul 6;19(7):e3001323. doi: 10.1371/journal.pbio.3001323 (PMC8284830; doi:10.1371/journal.pbio.3001323)
Supplement: S2 Table — (DOCX) [file pbio.3001323.s010.docx]

**S2 Table. Sequence of primers for qRT-PCR analysis.**

| Gene | Sequence (5' to 3') |
| --- | --- |
| *SYT7* | Forward: 5’-GCTGCTCTTGTCCCTCTGCTAC-3’ |
|  | Reverse: 5’-CATGGCTTTGAGGTTTCGAGCTTT-3’ |
| *DOC2A* | Forward: 5’-GGCGATCGCATGACCATCAA-3’ |
|  | Reverse: 5’-GGGAAGTAGTCGGAGATCTGG-3’ |
| *DOC2B* | Forward: 5’-TCATCGGTGGTGTGGTTCTG-3’ |
|  | Reverse: 5’-TGCCAACGCTCAATCCTCT-3’ |
| *SYT1* | Forward: TGAGACAAAAGTCCACCGGA |
|  | Reverse: GCCAAAATCCACGGTGTTCA |
